# Extracellular Vesicles in Human Preterm Colostrum Inhibit Infection by Human Cytomegalovirus In Vitro

**DOI:** 10.3390/microorganisms8071087

**Published:** 2020-07-21

**Authors:** Manuela Donalisio, Simona Cirrincione, Massimo Rittà, Cristina Lamberti, Andrea Civra, Rachele Francese, Paola Tonetto, Stefano Sottemano, Marcello Manfredi, Annalisa Lorenzato, Guido E. Moro, Marzia Giribaldi, Laura Cavallarin, Maria Gabriella Giuffrida, Enrico Bertino, Alessandra Coscia, David Lembo

**Affiliations:** 1 Laboratory of Molecular Virology, Department of Clinical and Biological Sciences, University of Turin, 10043 Orbassano, Italy; massimo.ritta@unito.it (M.R.); andrea.civra@unito.it (A.C.); rachele.francese@unito.it (R.F.); david.lembo@unito.it (D.L.); 2 Consiglio Nazionale delle Ricerche-Istituto di Scienze delle Produzioni Alimentari, 10095 Grugliasco (TO), Italy; simona.cirrincione@ispa.cnr.it (S.C.); cristina.lamberti@ispa.cnr.it (C.L.); laura.cavallarin@ispa.cnr.it (L.C.); gabriella.giuffrida@ispa.cnr.it (M.G.G.); 3 Neonatal Intensive Care Unit, Department of Public Health and Pediatrics, University of Turin, 10126 Torino, Italy; paola.tonetto@unito.it (P.T.); stefano.sottemano@unito.it (S.S.); enrico.bertino@unito.it (E.B.); 4 Center for Translational Research on Autoimmune and Allergic Disease (CAAD), University of Piemonte Orientale, 28100 Novara, Italy; marcello.manfredi@uniupo.it; 5 Candiolo Cancer Institute, FPO-IRCCS, 10060 Candiolo (TO), Italy; annalisa.lorenzato@unito.it; 6 Department of Oncology, University of Turin, 10060 Candiolo (TO), Italy; 7 Italian Association of Human Milk Banks, 20126 Milano, Italy; guidoemoro@tiscali.it; 8 Research Centre for Engineering and Agro-food Processing (CREA), 10135 Torino, Italy; marzia.giribaldi@crea.gov.it

**Keywords:** HCMV, breastfeeding, preterm, colostrum, extracellular vesicles, antiviral

## Abstract

Breast milk is a complex biofluid that nourishes infants, supports their growth and protects them from diseases. However, at the same time, breastfeeding is a transmission route for human cytomegalovirus (HCMV), with preterm infants being at a great risk of congenital disease. The discrepancy between high HCMV transmission rates and the few reported cases of infants with severe clinical illness is likely due to the protective effect of breast milk. The aim of this study was to investigate the anti-HCMV activity of human preterm colostrum and clarify the role of colostrum-derived extracellular vesicles (EVs). Preterm colostrum samples were collected and the EVs were purified and characterized. The in vitro anti-HCMV activity of both colostrum and EVs was tested against HCMV, and the viral replication step inhibited by colostrum-purified EVs was examined. We investigated the putative role EV surface proteins play in impairing HCMV infection using shaving experiments and proteomic analysis. The obtained results confirmed the antiviral action of colostrum against HCMV and demonstrated a remarkable antiviral activity of colostrum-derived EVs. Furthermore, we demonstrated that EVs impair the attachment of HCMV to cells, with EV surface proteins playing a role in mediating this action. These findings contribute to clarifying the mechanisms that underlie the protective role of human colostrum against HCMV infection.

## 1. Introduction

Human milk (HM) is considered the most important biofluid for the nutrition of infants and for the development of a child’s immune system by the World Health Organization [1]. HM provides protection against necrotizing enterocolitis, sepsis, enteric and respiratory infections and severe retinopathy and decreases the risk of death. For these reasons, the American Academy of Pediatrics recommends exclusive breastfeeding for the first 6 months of life, and colostrum is considered especially important for preterm newborns (up to one week postpartum) [2,3,4]. In spite of the well-known health benefits of HM, breastfeeding is also a common route for the mother-to-child transmission of human cytomegalovirus (HCMV), particularly in populations with a high HCMV seroprevalence. During lactation, the virus is shed into HM by almost every seropositive woman; maternal HCMV reactivation can already be detected in the colostrum and normally ends about three months after birth, as evidenced by PCR and viral culture analyses [5,6]. The postnatally acquired symptomatic HCMV infection rate is low in term infants, whereas preterm infants, especially those with a birth weight below 1500 g or those of less than 32 weeks of gestational age, are at the greatest risk of developing congenital disease through breastfeeding [5]. Systematic reviews have shown that breast milk-acquired HCMV infection with symptomatic disease is relatively rare [7,8]. The discrepancy of high HCMV transmission rates and the low number of reported infants with severe clinical illness are probably due to the protective effect of the components of breast milk against viral infection. We have recently demonstrated, in the context of preterm infants, that HM is endowed with intrinsic anti-HCMV properties, and their potency may vary according to the stage of lactation and the serological status of the mother [9]. Passive immunity is provided to newborns through a large number of soluble and cellular immune HM components, such as secretory immunoglobulins and leukocytes, as well as antimicrobial factors [10]. In this context, immunomodulatory and specific bioactive factors, such as lactoferrin, vitamin A and monolaurin, have been demonstrated to endow specific anti-HCMV properties. [11,12,13].

Extracellular vesicles (EVs) from human milk have recently become a subject of increasing interest for their protective role for infants [14]. EVs are complex structures, ranging from 50 to 300 nm in diameter, with an endosome-derived limiting membrane, secreted by a diverse range of cell types and found in all body fluids [15]. Milk EVs may originate from breast epithelial cells and from the macrophages and lymphocytes present in breast milk [16]. EVs have been found to be involved in cell–cell communication through the transfer of their cargos, which consist of microRNAs, noncoding RNAs, lipids and proteins from donor cells to recipient cells. Several current publications suggest an immune-regulatory role of milk EVs, especially exosomes, in both immune stimulation and toleration, and several studies have suggested their potential in immunotherapy [17,18]. Recent publications on the antiviral properties of EVs support the hypothesis that milk EVs are involved in the instruction of the protective role of the neonatal immune system [19]. In the present study, we have addressed the question of whether breast milk-derived EVs could inhibit HCMV infection, and we have explored how EVs can affect the viral replicative cycle steps. Furthermore, after conducting “membrane shaving” experiments, the antiviral activity of EVs was explored and a proteomic analysis of the shaved peptides was performed with the aim of characterizing the potential molecular players of antiviral activity.

## 2. Materials and Methods 

### 2.1. Human Colostrum Collection

Colostrum samples (1–5 days postpartum, 15 mL each) were obtained from 13 healthy mothers admitted to the Sant’Anna Hospital (Città della Salute e della Scienza di Torino, Turin, Italy) for preterm delivery between February 2017 and January 2020. An ethical review process was not required for this study since it was not a clinical trial. Each milk donor involved in this research signed a written consent form in which the data protection of the mother and infant was assured. Moreover, the donors were informed that only milk samples stored in excess would be used for research purposes, and the study design was explained. The donors cleaned their hands and breasts according to the Italian Human Milk Bank (HMB) guidelines [20]. Samples were collected, by means of an electric breast pump, in disposable sterile polypropylene Bisphenol-free bottles, to minimize the possibility of contamination. The main clinical characteristics of the study population are reported in Table 1.

### 2.2. Colostrum Clarification and Extracellular Vesicle (EV) Isolation

Ten-milliliter colostrum samples were centrifuged at 2000× *g* for 10 min, and the defatted colostrum was transferred to a new tube and centrifuged at 12,000× *g* for 30 min. The aqueous supernatant fractions were filtered (0.45 µm pore size filter), and two volumes of filtered supernatants were incubated overnight with one volume of Exoquick Exosome Precipitation Solution (SBI, System Biosciences, Palo Alto, CA, USA) at 4 °C. The following day, the mixture was centrifuged at 1500 g for 30 min at 4 °C, and the precipitated EVs were resuspended in PBS. The protein concentration was determined by means of a Protein Assay (Bio-Rad, Hercules, CA, USA).

### 2.3. EV Characterization by Immunoblotting

The protein profile of the EVs was determined by immunoblotting, using human foreskin fibroblasts (HFF-1) as a control. The EV preparations and HFF-1 cells were lysed in an RIPA buffer (25 mM Tris-HCl pH 7.6, 150 mM NaCl, 1% NP-40, 1% sodium deoxycholate, 0.1% sodium dodecyl sulfate (SDS) with a protease inhibitor cocktail. The supernatants were analyzed for protein concentrations by means of the Bio-Rad Protein Assay (Bio-Rad). The extracted proteins were denatured in a Laemmli buffer (4% SDS, 20% glycerol, 10% β-mercaptoethanol, 0.004% bromophenol blue, 0.125 M tris-HCl pH 6.8) at 95 °C for 5 min. The lysates were fractionated onto 8.5% SDS-PAGE and transferred to a polyvinilidene difluoride membrane (Millipore, USA). The following primary antibodies were used: CD9, CD63, CD81 (1:1000; SBI System Biosciences) and calnexin (1:1000, Transduction Laboratories, San Jose, CA, USA).

### 2.4. Nanoparticle Tracking Analysis (NTA)

A nanoparticle tracking analysis system (NanoSight NS300, Malvern Instruments Ltd, Malvern, UK) was used to determine the particle size and particle concentration. EV preparations were diluted in PBS (1:8000) prior to analysis and quantified in triplicate. Data were reported as the number of particles/mL (n/mL).

### 2.5. Cell lines and Viruses

Low-passage-number (<30) HFF-1 (ATCC SCRC-1041) were grown as monolayers in Dulbecco’s modified Eagle’s medium (DMEM) (Sigma-Aldrich, Sain Louis, USA), supplemented with 15% heat inactivated fetal bovine serum (FBS) (Sigma-Aldrich) and a 1% antibiotic solution (penicillin–streptomycin, Sigma-Aldrich). A bacterial artificial chromosome-derived HCMV Towne strain, incorporating the green fluorescent protein (GFP) sequence, was propagated on HFF-1 [21]. HCMV Towne titers were determined on the HFF-1 cells by means of a fluorescence focus assay, as previously reported [9]. The HCMV AD169 laboratory strain (ATCC VR-538) was propagated on HFF-1 cells by infecting freshly prepared confluent monolayers in DMEM 2% FBS. When the whole monolayer displayed a clear cytopathic effect, the infected cell suspension was collected, and the viral supernatant was clarified by means of centrifugation. Viral stocks were aliquoted and stored at −80 °C. HCMV-AD169 titers were determined on the HFF-1 cells using the median tissue culture infective dose (TCID_50_) method.

### 2.6. Cell Viability Assay

Colostrum samples and extracellular vesicles were investigated for their impact on cell viability by means of a 3-(4,5-dimethylthiazol-2-yl)-5-(3-carboxymethoxyphenyl)-2-(4-sulfophenyl)-2H-tetrazolium (MTS) assay, as described in Cagno et al. (2015) [22]. HFF-1 cells, pre-seeded at a 5 × 10^3^/well density in 96-well plates in DMEM 10% FBS, were challenged with serial dilutions of the aqueous fraction of colostra and extracellular vesicles. After 5 days of incubation at 37 °C, the cell monolayers were washed three times with DMEM, and cell viability was assessed with a Cell Titer 96 Proliferation Assay Kit (Promega, Madison, WI, USA). An aliquot of 20 microliters of MTS reagent was added to 100 µL DMEM per well; the cells were incubated at 37 °C for 4 h. Absorbance was measured at 491 nm using a Multiskan FC Microplate Photometer (Thermo Scientific, Waltham, MA, USA). The percentage of absorbances of the treated cells to the cells incubated with only a culture medium was calculated, and the 50% cytotoxic concentrations (CC_50_) and 95% confidence intervals (95% CI) were determined for the preterm colostra and colostrum-derived EVs using Prism software (Graph-Pad Software, San Diego, CA, USA).

### 2.7. HCMV Inhibition Assay

Pre-seeded HFF-1 cells (density 5 × 10^3^ per well in 96-multiwell plates) were pre-incubated with serial dilutions of colostra (1:1 to 1:1024) and EVs (from 423 µg/mL to 0.41 µg/mL) for 1 h at 37 °C. Corresponding serial dilutions of colostra and EVs containing a constant amount of HCMV Towne at a multiplicity of infection (MOI) of 0.02 foci forming units (FFU)/cell or HCMV AD169 at a MOI of 0.03 plaque forming units (PFU)/cell were incubated for 1 h at 37 °C and were then added to pre-treated HFF-1 cells. Viral adsorption was performed for 2 h at 37 °C. The inocula were removed; then, the cells were washed and were then overlaid with a 1.2% methylcellulose DMEM medium 2% FBS. After 5 days of incubation at 37 °C, the HCMV Towne-infected cells and foci were visualized as green fibroblasts by means of fluorescence microscopy and counted. The results were reported as the percentage of fluorescent cells compared to the control. The HCMV AD169-infected cell monolayers, after 5 days of incubation at 37 °C, were fixed with cold methanol-acetone (1:1) for 1 min, permeabilized in PBS 0.1 % Triton X-100, treated with PBS–methanol (1:1) 0.6% H_2_O_2_ and subjected to HCMV-specific immunostaining with a specific anti-HCMV IEA monoclonal antibody (1:1500 dilution; 11-003, Argene, Verniolle, France). An UltraTech HRP Streptavidin–Biotin detection system was used (Beckman Coulter, Marseille, France). The immunostained cells and plaques were counted, and the percentage of the inhibition of virus infectivity was determined. The concentrations of colostra and EVs that produced a reduction of 50% and of 90% in the formation of HCMV Towne foci (effective concentration-50 (EC_50_) and effective concentration-90 (EC_90_)) and of HCMV AD169 infectivity, in comparison with the controls, were calculated by means of Prism 5 software.

### 2.8. HCMV Inactivation Assay

Constant amounts of HCMV Towne (10^5^ FFUs) were challenged with EVs at EC_90_ concentrations (as determined in the inhibition assay) in a final volume of 110 µL. The virus–EV mixtures were incubated for 2 h at 37 °C. Both EV-treated and untreated HCMV mixtures were titrated to non-inhibitory concentrations on HFF-1 cells (density 5 × 10^3^/well) by means of a foci assay. Viral infectivity was determined after 5 days of incubation at 37 °C by means of fluorescence microscopy. The titers of the EV-treated and untreated viral mixtures were analyzed by means of the Student’s *t*-test. Significance was reported for *p* < 0.05.

### 2.9. Pre-Treatment Assay

HFF-1 cells (5 × 10^3^/well density) were challenged with serial dilutions of EV preparations at 37 °C for 2 h. After washing, the cells were infected with HCMV Towne at a MOI of 0.02 FFU per cell for 2 h, washed and incubated with a 1.2% methylcellulose DMEM medium with 2% FBS. After 5 days of incubation at 37 °C, the infected cells and foci were visualized by means of fluorescence microscopy and counted. The EC_50_ values of the EV preparations were calculated using Prism 5 software. 

### 2.10. Attachment Assay

Pre-chilled HFF-1 cells (5 × 10^3^/well density) were infected with HCMV Towne at a MOI of 0.2 (FFU per cell) at 4 °C in the presence of serial dilutions of EV preparations. After one wash, to remove the unbound virus, the cells were shifted to 37 °C for 2 h. The outer virions were inactivated with acidic glycine (0.1 M glycine, 0.14 M NaCl, pH 3) for 2 min at RT. The cells were then washed three times, overlaid with a 1.2% methylcellulose DMEM medium 2% FBS and incubated at 37 °C for 5 days. The infected cells and foci were visualized by means of fluorescence microscopy and counted. The EC_50_ values of the EV preparations were calculated using Prism 5 software. All the experiments were performed in triplicate.

### 2.11. Entry Assay

HCMV Towne, at a MOI of 0.2 FFU/cell, was adsorbed for 2 h at 4 °C on pre-chilled HFF-1 cells (5 × 10^3^/well density). After one wash to remove any unbound viral particles, the cells were treated with serial dilutions of EV preparations for 2 h at 37 °C. The outer virions were inactivated with acidic glycine for 2 min at RT. The cells were then washed three times, overlaid with a 1.2% methylcellulose DMEM medium 2% FBS and incubated at 37 °C for 5 days. The infected cells and foci were visualized by means of fluorescence microscopy and counted. The EC_50_ values of the EV preparations were calculated using Prism 5 software. All the experiments were performed in triplicate.

### 2.12. Post-Entry Assay

HCMV Towne, at a MOI of 0.2 FFU/cell, was adsorbed for 2 h at 4 °C on pre-chilled HFF-1 cells (5 × 10^3^/well density). After one wash, the cells were shifted to 37 °C for 2 h. The outer virions were inactivated with acidic glycine for 2 min at RT. The cells were then washed three times, challenged with serial dilutions of EV preparations in a 1.2% methylcellulose DMEM medium 2% FBS and incubated at 37 °C for 5 days. The infected cells and foci were visualized by means of fluorescence microscopy and counted. The EC_50_ values of the EV preparations were calculated using Prism 5 software. All the experiments were performed in triplicate.

### 2.13. Data Analysis

The EC_50_ and EC_90_ concentrations were calculated by means of regression analysis and compared using the F-test by Prism 5 Software. The Student’s *t*-test was used for the virus inactivation assay to compare the viral titers in the EV-treated and untreated samples, as well as for the attachment assay to compare the number of HCMV foci. A one-way ANOVA was used to compare HCMV infectivity in the attachment assays with the pooled peptides. Significance was reported for *p*< 0.05. All the experiments are presented and discussed as the means of the results of three independent experiments performed in duplicate.

### 2.14. Shaving of Extracellular Vesicles

An aliquot of 100 µL of EVs, resuspended in PBS, was mixed with an equal volume of 25 mM NH_4_HCO_3_. An aliquot of 3 µL of trypsin (1 µg/µL) (Promega, Madison, WI, USA) was added and the sample was incubated at 37 °C for 4 h under mild agitation in order to allow surface protein digestion. The shaved EVs were washed with sterile PBS using a 300kDa Nanosep Centrifugal Device (Pall Corporation, New York, US) previously passivated for 1 h with 5% Tween80 and washed once with 70% ethanol, once with sterile ddH_2_O and twice with 25 mM NH_4_HCO_3_. In order to obtain the tryptic peptide fraction, the solution that passed through the membrane was collected and further filtrated with 10kDa Nanosep. The peptides were then dried under vacuum (Concentrator 5301, Eppendorf AG, Hamburg, Germany) before LC–MS/MS analysis. As a control, the EV preparation was processed, as already described, without the trypsin digestion step.

### 2.15. ESI-Q-TOF Analysis of Peptides

LC–MS/MS analyses were performed using a micro-LC Eksigent Technologies (Dublin, USA) system with a stationary phase of a Halo Fused C18 column (0.5 × 100 mm, 2.7 μm; Eksigent Technologies, Dublin, USA). The injection volume was 4.0 μL and the oven temperature was set at 40 °C. The mobile phase was a mixture of 0.1% (v/v) formic acid in water (A) and 0.1% (v/v) formic acid in acetonitrile (B), which was eluted at a flow-rate of 15.0 μL/min at increasing concentrations of B; that is, from 2% to 40% in 30 min. Peptides were suspended in 10 μL of A. The LC system was interfaced with a 5600+ TripleTOF system (AB Sciex, Concord, Canada), equipped with a DuoSpray Ion Source and CDS (Calibrant Delivery System). Peptide identification was performed using the traditional data-dependent acquisition (DDA) method. The MS data were acquired with Analyst TF 1.7 (SCIEX, Concord, Canada).

### 2.16. Peptide Data Search

The MS files were searched using Mascot v. 2.4 software (Matrix Science Inc., Boston, MA, USA) with trypsin as the enzyme, a peptide mass tolerance of 50 ppm, an MS/MS tolerance of 0.1 Da, peptide charges set to 2 ^+^, 3 ^+^ and 4 ^+^ and under consideration of the monoisotopic mass. The UniProtKB review database containing human proteins (Human Uniprot, version v.2018.02.01, 42,271 sequences) was used. Only the peptides with a higher peptide score than the peptide identity were considered for the protein identification. Moreover, only the proteins identified in at least two samples out of three were selected as shared proteins and subjected to further in silico analyses. In the case of common proteins identified with a single peptide, they were only included if they were identified with at least two peptides in another sample containing the same protein.

### 2.17. In Silico Analysis of Proteins

The 45 proteins selected as previously described were analyzed using three open-source tools. Cell Plock 2.0 (package Euk-mPLoc 2.0) (http://www.csbio.sjtu.edu.cn/bioinf/euk-multi-2/) was used to find the protein sub-localization, Protter version 1.0 (http://wlab.ethz.ch/protter/start/) was used to visualize the protein arrangement between the internal and external environment of the cell, and InterPro (https://www.ebi.ac.uk/interpro/) was used to classify the proteins according to the biological process in which they are involved.

## 3. Results and Discussion

### 3.1. Milk Sample Collection and Antiviral Activity of Colostrum-Derived Extracellular Vesicles (EVs) against HCMV

**Figure 1:**
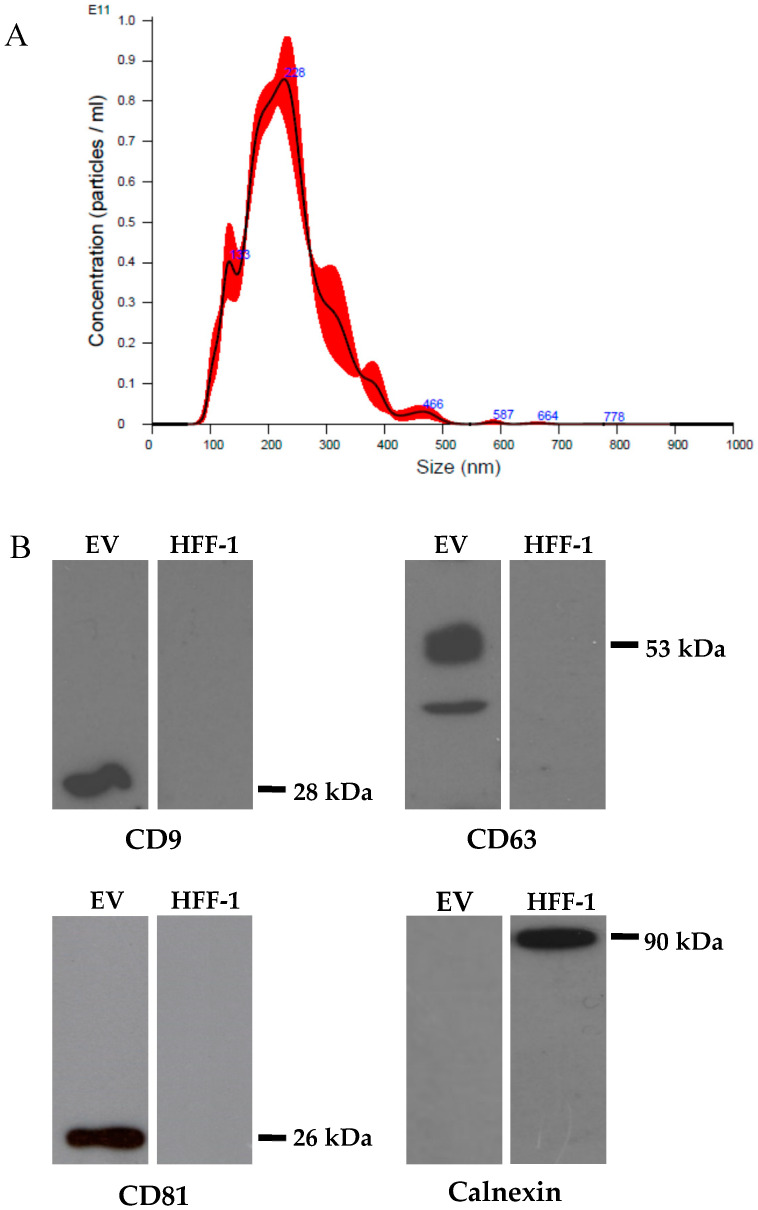
Characterization of the breast milk extracellular vesicles (EVs). (**A**): Representative analysis of an EV sample obtained from nanoparticle tracking analysis (NTA) using NanoSight. Size (nm) on the X-axis and concentration (number of particles/mL) on the Y-axis. The NTA showed that most EVs were between 100–400 nm in diameter (mean size, 219.9 ± 28.7 nm, mode 184.4 nm). (**B**): Profile of the colostrum-derived EVs obtained from immunoblotting. The CD9, CD63 and CD81 EV markers and the endoplasmic reticulum-related protein calnexin were analyzed. Human foreskin fibroblast (HFF-1) cell lysate was used as a control. The immunoblotting revealed that the colostrum-derived EVs were positive for CD63, CD9 and CD81 and negative for calnexin.

The first set of experiments was performed on 13 fresh colostrum samples (1–5 days postpartum) collected from healthy donor mothers admitted to the Sant’Anna Hospital of Turin (Città della Salute e della Scienza di Torino). This study group included mothers of preterm infants with gestational ages ranging from 23 + 3 to 32 + 0 (weeks + day). The milk was collected in the hospital and kept at room temperature until it was delivered to the laboratory, where it was processed within two hours. The aqueous fraction derived from clarified colostrum is considered the most appropriate biological matrix for in vitro assays because of its lower impact on cell viability than whole milk [9]; it was therefore used as the starting material for the isolation of EVs with an ExoQuick preparation [23,24]. The EV pellet was dissolved in a small volume of PBS, and its protein concentration was determined. Vesicles purified from preterm colostra were characterized using nanoparticle tracking analysis to determine the size and particle concentration. The Nanosight instrument showed that most EVs were between 100–400 nm in diameter (mean size, 219.9 ± 28.7 nm, mode 184.4 nm; a representative analysis is reported in Figure 1A). The particle concentration of the milk EVs was roughly 1.00 × 10^13^ particles/mL. Minimal differences in size and concentration were observed for the different EV preparations. Protein lysates were prepared and Western blot analysis was performed to confirm the presence of exosomes in the EVs. The immunoblotting revealed that the colostrum-derived vesicles were positive for the CD63, CD9 and CD81 exosome-associated proteins, but negative for calnexin, which was only detected in the HFF-1 cell lysate. The negative calnexin signal indicated that the EVs did not contain an endoplasmic reticulum marker (Figure 1B) [25,26].

After the isolation and characterization of the EVs, colostrum samples and the corresponding isolated EVs were tested in vitro on HFF-1 cells against HCMV Towne strain expressing the GFP protein; the GFP marker facilitated the identification of infected cells. As reported in Table 2, we confirmed the net antiviral activity of colostrum against HCMV, with the presence of only moderate differences among samples; all the tested samples generated dose–response curves with a half maximal effective concentration (EC_50_ value) ranging from 38.71 µg protein/mL to 213.9 μg protein/mL. The observed variability in the anti-HCMV activity of human colostrum was likely related to the well-known interpersonal variability of HM composition and to the HCMV serostatus of the milk donors. Furthermore, we demonstrated a remarkable antiviral activity in all the EV preparations, with EC_50_s ranging from 3.98 to 75.93 µg protein/mL.

**Figure 2:**
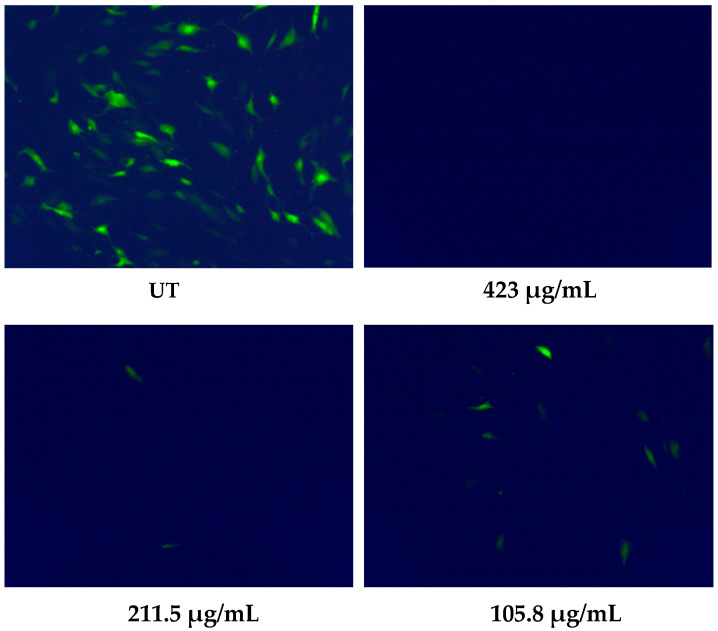
Anti-HCMV assay with colostrum-derived EVs. Representative HCMV foci (green fluorescent protein (GFP)-expressing, green) in the HFF-1 cell monolayers are reported for 423, 211.5 and 105.8 µg/mL EV protein concentrations, as obtained from the HCMV Towne inhibition assay; HCMV at MOI 0.3 FFU/cell. UT, untreated.

Figure 2 shows that a high number of GFP-expressing cells were visible in the untreated infected monolayers. On the other hand, the viral infectivity was completely abolished for the highest tested dose (423 µg/mL), and a reduced number of green cells was reported for lower EV doses. Interestingly, a high level of antiviral activity of the colostrum-derived EVs (an EC_50_ value of 41.56 µg protein/mL) was observed, even when a 10-fold greater viral inoculum was used (sample 3). Moreover, we excluded the possibility that the antiviral activity of the EVs was due to cytotoxicity, since the EVs did not affect the cellular viability when the cells were treated under the same conditions as the antiviral assays and processed by means of an MTS assay (CC_50_ values > 1000 µg/mL for all the EV preparations). Figure S1 reports cell viability data from assays performed on three representative EV preparations (EV samples 1, 2 and 3).

To the best of our knowledge, this is the first study to have reported the intrinsic antiviral activity of human colostrum-derived EVs against HCMV infection. Näslund et al. (2014) demonstrated a similar antiviral activity of breast milk exosomes and showed that milk exosomes, but not plasma exosomes, significantly reduced the productive HIV-1 infection of monocyte-derived dendritic cells by approximately 50%, compared with untreated cells, and blocked HIV-1 transfer from monocyte-derived dendritic cells to CD4+ T cells [19]. Only a few studies have reported a direct inhibition of viral infections by the extracellular vesicles of biological fluids. Extracellular vesicles isolated from semen, but not from the blood of HIV-1–infected individuals, inhibited HIV-1 replication in vitro [27]. Furthermore, exosome-like vesicles released from human tracheobronchial ciliated epithelium neutralized the infectivity of the influenza virus by up to 85–99% [28].

### 3.2. Colostrum-Derived Extracellular Vesicles Inhibit the Attachment of HCMV on Cells

**Figure 3:**
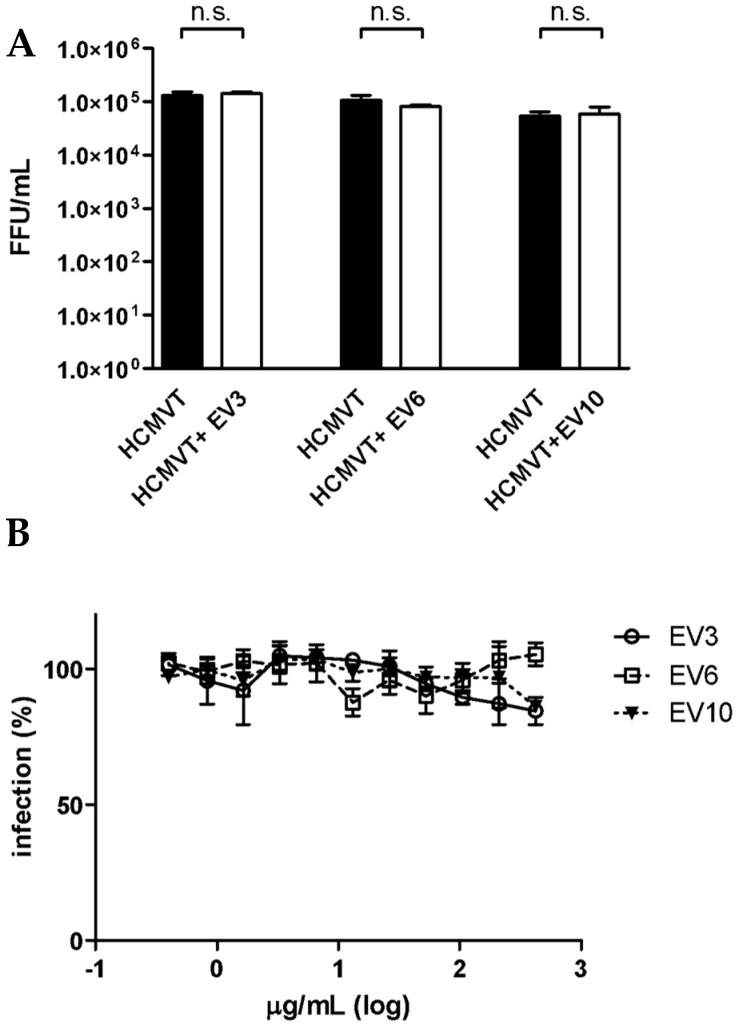
(**A**). Evaluation of the inactivation of HCMV by EVs. Three samples of EVs were investigated to evaluate the inactivation of the HCMV particles at 37 °C for 2 h. No significant differences in the viral titers were observed between the treated and untreated samples. The infectious titers are expressed on the Y-axis as FFU/mL. Black bar, HCMV; white bar, HCMV plus EV sample. Error bars represent the standard error of the means of three independent experiments. (**B**). Pre-treatment anti-HCMV assay. Three samples of EVs were challenged with HFF-1 cells for 2 h at 37 °C prior to HCMV infection. None of the EV preparations exerted inhibitory activity under these experimental conditions. Data are reported as the mean percentages of infection, in comparison with the control of three independent experiments.

In order to identify the major mechanism of action, three EV preparations (3, 6 and 10) were selected and subjected to further studies. First, a virus inactivation assay was performed in which the virus was incubated with an effective high concentration of EVs (EC_90_) for 2 h at 37 °C and then added to cells at dilutions at which the vesicles were no longer active. Figure 3A shows no significant differences in the viral titers between the treated and untreated samples, indicating that the vesicles did not impair infectivity by directly targeting viral particles. Having excluded virus inactivation activity, we then investigated whether the active EVs targeted the cell surface before the infection, thereby inhibiting infectivity. A pre-treatment assay was performed in which Vero cells were pre-treated with scalar protein concentrations of EVs for two hours prior to viral infection. Figure 3B shows that none of the tested EV preparations exerted inhibitory activity under these experimental conditions. 

**Figure 4:**
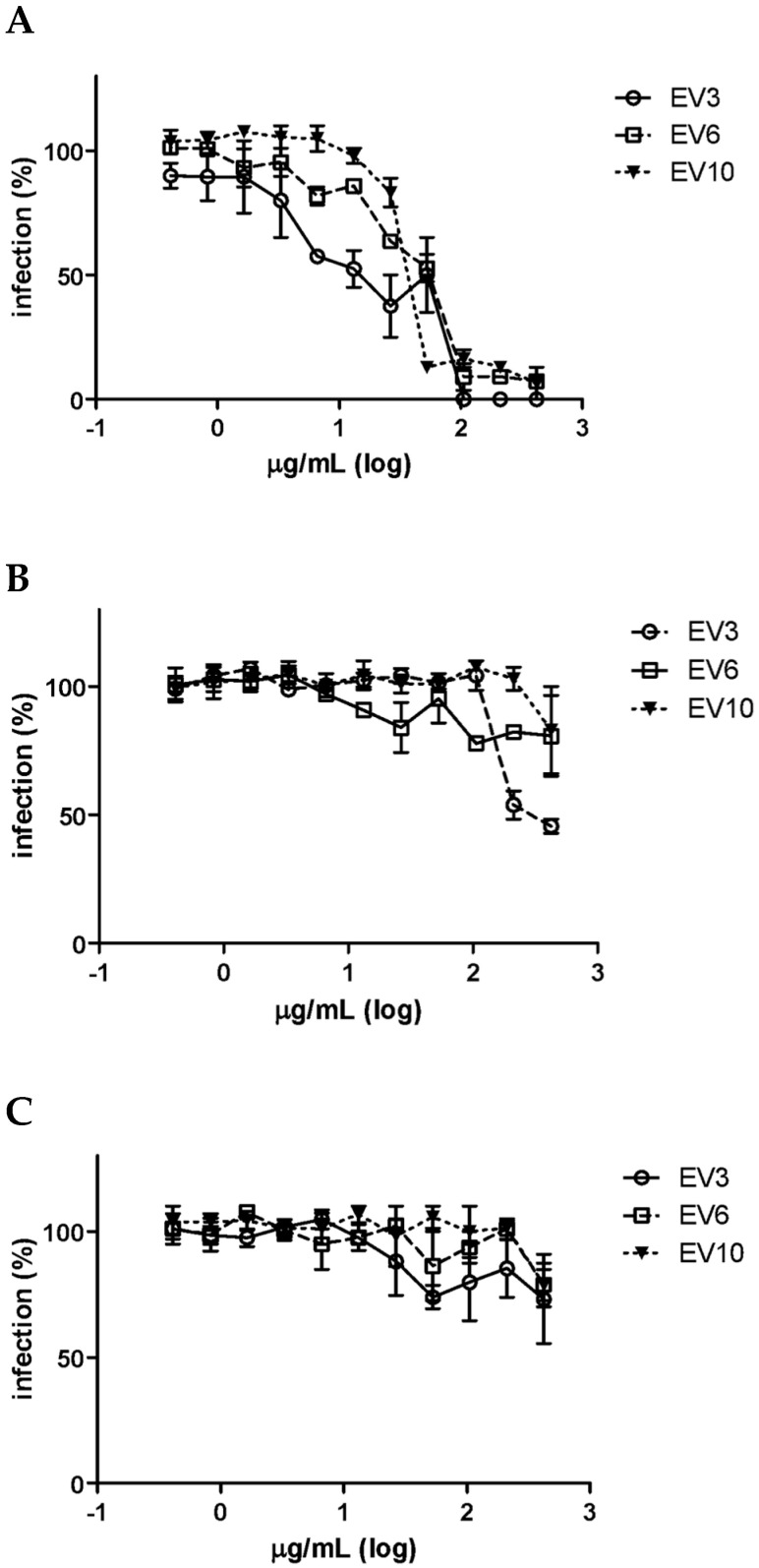
Effect of the colostrum-derived EV on the HCMV replicative cycle. (**A**) of the above figure shows the attachment assay; in this experimental condition, the virus was allowed to bind to the surface of the HFF-1 cells in the presence of EV but not to undergo cell entry. All the EV preparations inhibited HCMV Towne infectivity and generated dose–response curves. (**B**) shows the entry assay; in this experimental condition, the EVs were added to the HFF-1 cells immediately after virus attachment. No inhibition was observed for EV preparations 6 and 10, and a weak inhibition was observed for high doses for EV sample 3. (**C**) shows the post-treatment assay; the EVs were added to HFF-1 cells immediately after the viral penetration of the cells. No inhibition of HCMV infectivity was observed at the post-entry stage of infection for any of the EV preparations. Data are reported as the mean percentages of infection, compared with the control, for three independent experiments and for three EV preparations.

We then evaluated the ability of EVs to impair the first steps of the HCMV replicative cycle, such as their attachment and entry into cells, by means of specific assays. First, we carried out an attachment assay, using an experimental condition in which the virus was allowed to bind to the surface of the host cells, in the presence or absence of EVs, but did not undergo cell entry. Figure 4A shows that all the EV preparations inhibited HCMV infectivity and generated dose–response curves with EC_50_ values ranging from 14.50 to 38.90 μg protein/mL. It should be highlighted that no statistically significant differences were observed for any of the investigated EV preparations (*p* > 0.05, Student’s *t*-test) in the comparison between the number of HCMV foci in the untreated wells and those in the treated wells, with no active EV dilutions in the attachment assays, thus suggesting that the viral titers did not change. On the other hand, when EVs were added immediately after virus attachment to assess their ability to prevent entry (entry assay), no inhibition was observed for two EV preparations, and a weak inhibition was observed at high doses for the sample 3-derived EVs (Figure 4B). Furthermore, in order to exclude the ability of EVs to impair the late intracellular steps of viral replication, vesicles were added immediately after the viral penetration of the host cells. The post entry assay, which is reported in Figure 4C, showed no inhibition of HCMV infectivity at the post-entry stage for any of the tested EV preparations. 

Overall, these findings demonstrate that human colostrum-derived EVs exert their antiviral activity by impairing the attachment of HCMV to the cell surface. This effect may be attributed to several EV components acting together, or to a single species/component, likely including proteins, glycoproteins and lipids which are present on the EV surface. These antiviral effectors might act through two distinct approaches: first, by binding to specific viral components while mimicking cellular receptors, or through a direct interaction with the cell, and masking cellular receptors, thereby competitively inhibiting virus attachment. Like viruses, EVs can also bind to the plasma membranes of other cells, enter them through either fusion or endocytosis and trigger specific reactions. These processes are mediated by specific EV proteins, and tetraspanins in particular [29].

### 3.3. Shaving of Extracellular Vesicles Significantly Reduced Their Anti-HCMV Activity

**Figure 5:**
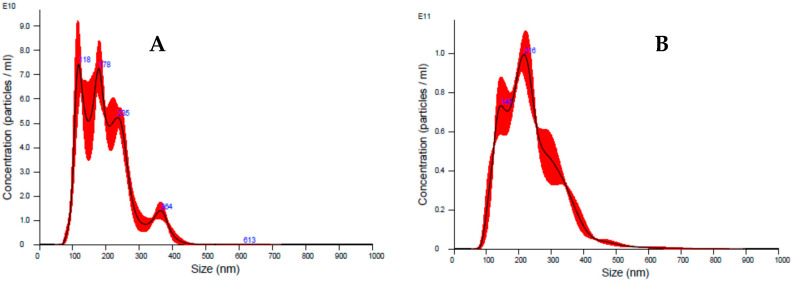
Characterization of shaved EVs. Representative analysis of a shaved EV sample (**A**), and intact EV (**B**) by means of nanoparticle tracking analysis (NTA) using NanoSight. The shaved EVs showed a reduction in diameter compared with the intact EVs. Size (nm) on the X-axis and concentration (number of particles/mL) on the Y-axis.

Shaving experiments were performed on three EV preparations (11, 12 and 13) to evaluate the ability of EVs to affect the interaction between the viral particles and the cell surface proteins. To this end, the EVs were subjected to trypsin digestion in order to shave the proteins that were present on the surface of the EVs. As a control, an aliquot of each EV preparation was subjected to the same shaving protocol but without digestion with trypsin. The shaved EVs were characterized using NanoSight to evaluate their size, which showed a reduction in diameter (mean size 188.2 nm, mode 138.9 nm) compared with intact EVs (mean size 219.9 nm, mode 184.4 nm) (Figure 5), while no diameter variations were reported for the control EVs. 

**Figure 6:**
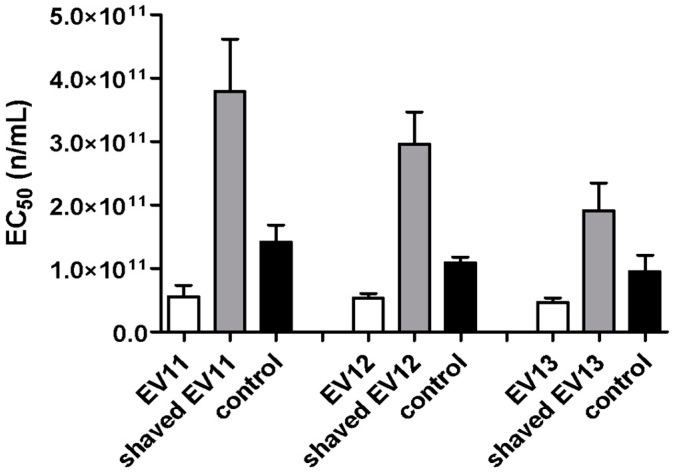
Anti-HCMV assays with EVs and shaved EVs. The antiviral activities of three EV preparations, shaved EVs and controls are reported as EC_50_ values obtained from an HCMV inhibition assay. The shaved EVs from three preparations (11, 12 and 13) exerted a significantly reduced antiviral activity compared with both the intact EV and the control preparations. The inhibition test data were normalized to the number of vesicles and expressed as the number of particles/mL (n/mL). Error bars represent the standard error of the means of three independent experiments.

After shaving, the expected decrease in the number of EVs was observed, and the inhibition test data were therefore normalized to the decreased number of vesicles. Antiviral assays against the HCMV Towne strain were then performed by treating cells with intact, shaved and control EVs. As reported in Figure 6, the shaved EVs of the three preparations exerted a significantly reduced antiviral activity, compared with both the intact EV and control preparations. The EC_50_ values of the intact EVs were around 3.65 × 10^10^ particles/mL, while the EC_50_ values of the shaved EVs were in a range from 1.47 × 10^11^ to 4.61 × 10^11^ particles/mL. In order to validate these data, antiviral experiments were also performed against HCMV strain AD169—another laboratory reference strain. Again, the clarified colostrum exhibited a net anti-HCMV-AD169 activity (EC_50_ 163 µg/mL), and the intact colostrum-derived EVs exerted a high antiviral activity (EC_50_ 24.2 µg/mL), which was significantly reduced after the shaving experiments (shaved EVs, EC_50_ 201 µg/mL). Our results indicate that the proteins on the surface of EVs play a role in the antiviral mechanism of action of EVs.

### 3.4. Proteomic Analysis of EV Surfaceome 

The shaved peptide mixture derived from the EV membrane surface proteins (surfaceome) was analyzed by means of LC–MS/MS in order to investigate the proteins that could be responsible for the EV antiviral properties (Table 3; more details are shown in Table S1).

**Figure 7:**
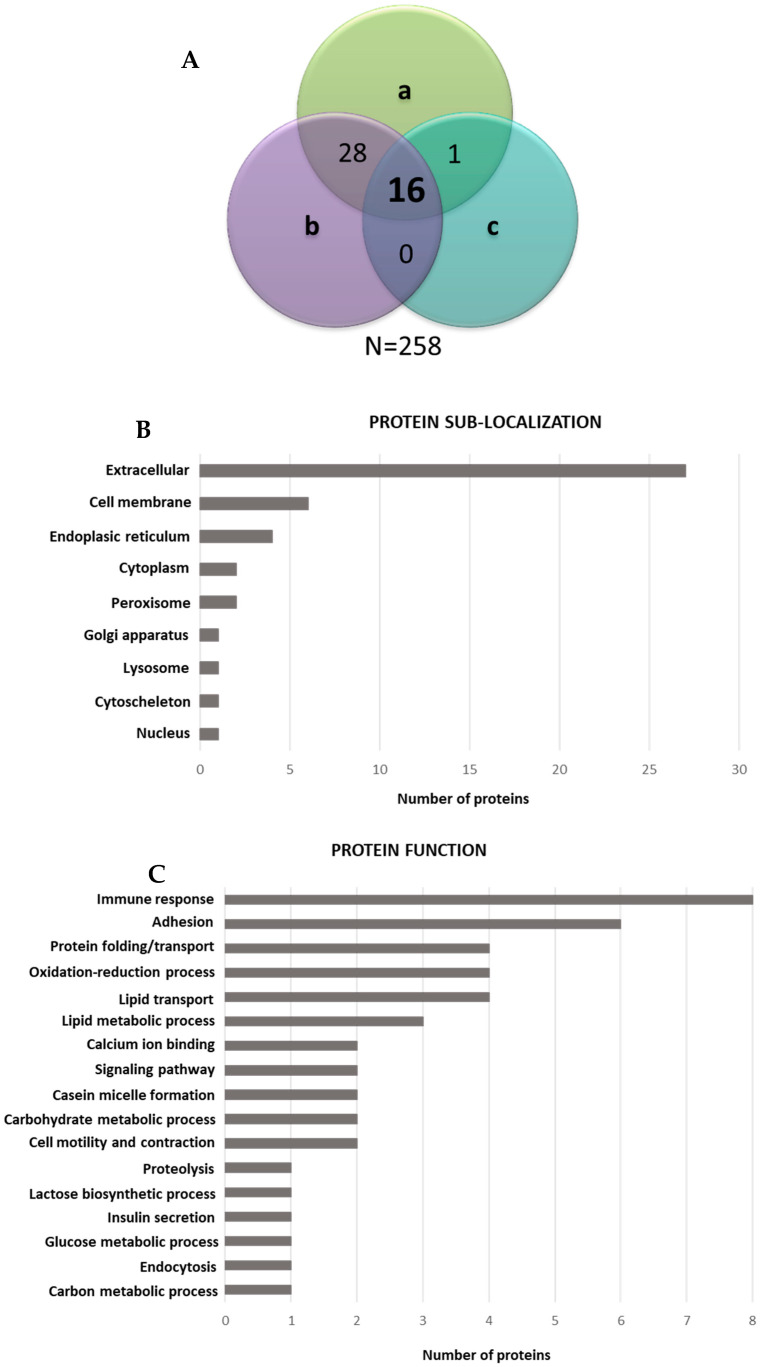
Venn diagrams showing the distribution of the identified proteins over samples 11 (a), 12 (b) and 13 (c). A total of 258 proteins were identified in all the samples (**A**). Histograms grouping the 45 shared proteins (identified in at least two out of the three analyzed samples) according to the sub-localization (**B**) and to the function (**C**).

The total number of identified proteins in the three analyzed samples was 258. Of these proteins, 45 were found to be shared between at least two samples out of three, and 16 were found in all the analyzed samples (Figure 7A). Sample 11 and sample 12 shared a higher number of proteins than sample 13, which seemed to be less rich in shaved peptides. Considering that only the proteins with an extracellular domain containing cutting sites for trypsin were identified, we believe that the number of identified proteins is adequate. As far as sub-localization is concerned, the extracellular proteins were most abundant, followed by the cell membrane proteins (Figure 7B). The analyses of the membrane protein coverage by the identified peptides showed that 98% of the peptides were located in the extracellular regions, thus demonstrating the effectiveness of the shaving protocol. The most frequently represented biological function categories were those related to the immune response and adhesion (Figure 7C), thus indicating that these are the key mechanisms involved in antiviral response.

The surfaceome proteomic analysis showed that 91% of the identified proteins had already been found in the HM EVs [17,30]. Some of them are already known for their antiviral properties, especially against HIV infection, whereas there are scarce data regarding their inhibitory effect on HCMV. Interaction with both the virus and the host cell receptors has been proposed as a mechanism of action for most of these proteins (e.g., clusterin, bile salt-activated lipase, tenascin, thrombospondin and osteopontin). Among the identified proteins, the most promising in terms of antiviral properties are components of the immune system (polymeric-Ig receptor precursor, Ig κ-chain C region, Ig heavy constant alpha 1 and complement C9 and C3), caseins (α-S1-casein, β-casein and k-casein), whey proteins (lactoferrin and α lactalbumin), milk fat globule-associated proteins (MFGP) (mucin MUC4, lactadherin, clusterin, bile salt-activated lipase, apolipoproteins and lipoprotein lipase), macrophage mannose receptor, tenascin, thrombospondin, gelsolin and osteopontin. 

The components of the immunoglobulin superfamily—and IgAs in particular—are already known to be prominent in human breast colostrum and to provide newborns with passive immune protection. Among the complement proteins, complement component C9—which has here been identified for the first time in the HM EV fraction—is the major actor of the membrane attack complex (MAC), a multi-protein complex that forms pores in the plasma membrane of target pathogens or virally infected cells [31]. C9 polymerization is the product of a single gene that is sufficient to encode a pore-forming molecule targeted at an early stage by C3b (also identified in this study). C9 is a multi-domain protein that contains an N-terminal type-1 thrombospondin (TSP) domain, an LDL-receptor class A repeat, a number of potential transmembrane regions and a C-terminal EGF-like domain. The N-terminal TSP1 domain seems to be crucial in the molecular mechanism of the MAC assembly. The formation of a MAC on the viral surface and complement-mediated lysis seem to be essential for the reduction of viral titers in Zika virus infection [32].

Most of the proteins identified in our study are phosphorylated and/or glycosylated and thus function as soluble receptors that inhibit pathogen binding to the mucosal cell surface. Caseins, for instance, can exert their antiviral activity with both phospho-groups and glycans. Κ-casein has been reported to have anti-human rotavirus activity via the direct binding of glycans to the virus [33]. Among the whey proteins, lactoferrin (LF) has a well-known antiviral activity against HCMV as well as against the hepatitis C virus, poliovirus, enterovirus 71, BK polyomavirus, HIV-1 and the human papilloma virus [34]. The cationic N-terminal amino acid sequence of both human and bovine LF, which are rich in arginine and tryptophan, contributes to anti-HCMV activity [35]. Lactoferricin, mainly in the cyclic structure, which is derived from the LF N-terminal part by proteolytic cleavage with pepsin, primarily exhibits its antiviral effect on the cell surface, where it interferes between the virus and host cell receptors and thus blocks HCMV adsorption and entry [36]. Alpha-lactalbumin has also demonstrated antiviral activity against reovirus strain type 3 Dearing, for which a hemagglutination mechanism has been suggested [37]. 

Regarding milk fat membrane associated proteins, the involvement of human milk mucins in protection against virus infection has already been established. Purified mucin components (MUC1 and MUC4) are able to inhibit HIV-1 in vitro and prevent the transmission of HIV-1 from dendritic cells to CD4+ T cells [38,39]. Moreover, core 2 glycans of mucins bind to the rotavirus virion, thereby preventing the virus from recognizing the cellular receptors [40]. Furthermore, the presence of epithelial mucins, associated with a-2,6-linked sialic acid, on exosome-like vesicles from human tracheobronchial ciliated epithelium has been shown to contribute to their antiviral effect against the human influenza A virus, which is known to bind sialic acid. The antiviral activity was ablated by pre-treating EVs with neuraminidase, thus suggesting that the interaction between exosomes and the influenza A virus was sialic acid-dependent [28]. Milk exosomes instead act as a protective factor against the vertical transmission of HIV-1 by competing with the virus to bind to the cellular receptor dendritic cell-specific ICAM-3–grabbing non-integrin (DC-SIGN). This action was found to be due to the presence of the soluble MUC 1 on the exosome surface—a known DC-SIGN ligand [19]. Among the MFGPs, lactadherin (LA) is a glycoprotein that is able to attenuate the infectivity of rotavirus by interacting directly between the virus and its oligosaccharides, thus preventing the virus from attaching to the intestinal mucosa [41]. As already demonstrated for MUC1, the N-linked carbohydrate sequence containing sialic acids is an essential feature due to its antiviral activity, and the removal of sialic acid in fact results in a loss of its inhibitory activity [42]. Another MFGP is clusterin; although it is ubiquitously expressed, it is engaged in a variety of physiologic and pathologic processes, including inflammation, atherosclerosis and cancer. It may display different biological functions according to its glycosylation pattern [43]. Sabatte et al. (2011) demonstrated that semen clusterin is a high-affinity ligand of the C-type lectin receptor DC-SIGN and is responsible for the complete inhibition of HIV-1 recognition [44]. DC-SIGN is expressed by immature monocyte-derived dendritic cells (MDDC) and macrophage subsets and binds specific carbohydrate structures expressed on the pathogen surface, thereby mediating the internalization of different pathogens, including HCMV [45]. Moreover, Tripathi et al. (2013) demonstrated an interaction between the influenza A virus nucleoprotein and the host antiapoptotic factor clusterin, which is involved in host cell death induction [46]. A few other viruses, including the dengue virus and hepatitis D, also target clusterin to modulate host responses [47,48]. Bile salt-activated lipase (BSSL), like clusterin, is able to bind DC-SIGN and cause the inhibition of the HIV-1 transfer to CD4+ T cells [49]. Moreover, BSSL is effective against the Norwalk virus, probably because its tandem repeat O-glycosylated motifs attract the virus by acting as decoy receptors [50].

The macrophage mannose receptor (MRC1)—a single-pass transmembrane glycoprotein belonging to the C-type lectin family—has been investigated in great detail. It is found on the surface of most tissues—macrophages, dendritic cells and some lymphatic or liver endothelial cells—where it plays the role of capturing pathogens and removing them from circulation in the bloodstream [51]. As already demonstrated by Taylor et al. (2005), MRC1 is able to recognize specific sugar motifs (i.e., mannose modification) on pathogen membrane proteins and to mediate pathogen uptake [52]. It is able to interact with different viruses (HIV-1, dengue, hepatitis B and influenza A). As recently reported by Sukegawa at al. (2018) with reference to HIV-1, MRC1 inhibits virus release and mediates HIV retention on the surface of virus-producing cells, thus causing a reduction in the progression of infection [53]. To the best of our knowledge, the possible involvement of MRC1 in the HCMV replication cycle has never been investigated. However, considering that MRC1 is expressed on the main target HCMV-infected cells, including macrophages and endothelial cells, its ability to bind viral glycoproteins and prevent cell surface attachment can be hypothesized. Further studies are required to confirm this mechanism of action. 

Thrombospondin (TSP1) is an extracellular matrix glycoprotein with functional domains that is able to bind calcium, heparin, collagens, other matrix components and cell surface receptors, including CD36, heparan sulfate proteoglycans (HSPG) and integrins. It has been demonstrated that HCMV infection suppresses the expression of TSP1 and 2 in human fibroblasts, in human retinal glial cells and in primary fetal astrocytes, and this action is mainly mediated by HCMV immediate–early proteins [54,55]. Considering the early steps of the HCMV replicative cycle, viral attachment involves envelope glycoproteins, such as glycoprotein M and B (gM and gB), both of which bind to HSPGs on the cell surface. The virus then moves quickly to a more stable cell type-specific docking interaction, with one or more cellular surface receptors, including specific integrins, epidermal growth factor receptor (EGFR) and platelet-derived growth factor receptor (PDGFR)-α, which mediate HCMV attachment and entry [56]. Therefore, given the ability of TSP to bind several cellular receptors, including HSPG, it is possible to hypothesize that it might be able to competitively inhibit virus attachment. 

Tenascin is a large, hexameric extracellular matrix glycoprotein that is heavily sialylated: each monomer contains an assembly domain, a region of epidermal growth factor-like repeats, a region of fibronectin type III-like domains and a fibrinogen-like globe [57]. Fouda et al. (2013) isolated tenascin from the HIV-neutralizing fraction of antibody-depleted breast milk [58]. It is able to mediate HIV-1 capture, to block virus-epithelial cell binding and to neutralize HIV-1 by binding to the HIV Envelope variable 3 loop in a charge-dependent manner, depending on the presence of sialic acid in its glycomoieties. Furthermore, Mangan et al. (2019) demonstrated that the interaction between the negatively charged fn domain of tenascin and the positively charged regions of the chemokine coreceptor binding site of HIV-Env is a likely mechanism of tenascin-mediated virus neutralization [59]. According to these data, it would be interesting to evaluate whether tenascin represents an innate mucosal antiviral factor that is present as an endogenous HCMV neutralizing protein in breast milk and to investigate its mechanism of action. 

Another protein involved in the anti-HIV-1 mechanism of action is gelsolin, which belongs to the actin binding protein superfamily and is known to promote actin cytoskeleton remodeling. García-Expósito et al. (2013) demonstrated that gelsolin can compromise HIV-induced actin reorganization and the viral receptor capping events required for HIV-1 Env-mediated fusion, entry and infection. Therefore, gelsolin can constitute a barrier that restricts the HIV-1 infection of CD4+ lymphocytes in a pre-fusion step [60]. 

Finally, osteopontin is a multifunctional protein that is involved in both innate and adaptive immunity. Zhao et al. (2016) demonstrated that osteopontin is an essential positive regulator that protects the host from virus infection through the stabilization of the tumor necrosis factor receptor (TNFR)-associated factor 3, induction of interferon regulatory factor 3 activation and interferon beta (IFN-β) production [61].

### 3.5. Surface Peptide Mixtures Inhibit HCMV Attachment

**Figure 8:**
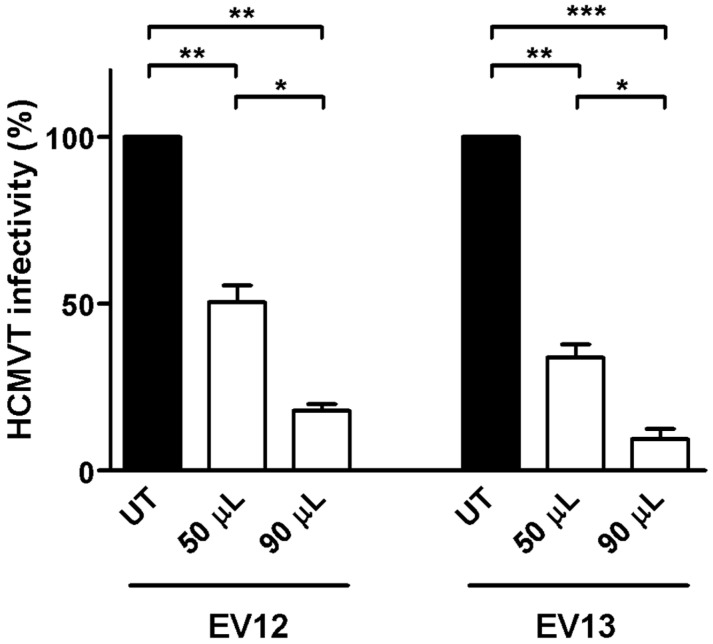
Effect of the peptides from shaved colostrum-derived EVs on the HCMV replicative cycle, as determined by means of the attachment assay. In this experimental condition, the virus was allowed to bind to the surface of HFF-1 cells in presence of EV but did not undergo cell entry. Peptides from both shaved EV preparations (12 and 13) were dissolved in 420 µL DMEM, and two different volumes were challenged. Both of the preparations inhibited the viral attachment in a dose–response manner in comparison with the untreated control. Data are reported as percentages of infection, in comparison with untreated controls for two shaved EV preparations, and compared by means of a one-way ANOVA (*, *p* < 0.05; **, *p* < 0.01; ***, *p* < 0.001). UT, untreated.

The peptide mixtures derived from the EV shaving of preparations 12 and 13 were investigated for their anti-HCMV Towne potency. Pooled peptides were dissolved in 420 µL DMEM medium, and two different volumes of the samples were assessed in triplicate by means of an attachment assay. As reported in Figure 8, a significant dose-dependent HCMV inhibition of the viral attachment was observed, with slight variability between the two peptide pools. The 90 µL volumes from EVs 12 and 13 reduced HCMV attachment by 82.04% and 90.46% in comparison with the untreated control, respectively. The 50 µL EV preparations inhibited the viral attachment in the 49.5–66.16% range in comparison with the untreated control. These data suggest the direct involvement of EV surface peptides in the inhibition of viral attachment to cells. The incomplete inhibition of viral attachment from peptide mixtures may be attributed to changes in the structural folding of surface proteins due to enzymatic digestion. Considering these data, the identification of peptides by means of LC–MS/MS and the in silico characterization of the peptides will be performed with the aim of characterizing the peptides that are potentially responsible for the observed antiviral activity. The most promising peptides will then be synthetized for further antiviral in vitro experiments.

## 4. Conclusions 

Overall, our results provide novel insights into the protective role of human colostrum against HCMV infection in reducing the risk of viral transmission from mother to child through breastfeeding. We have confirmed the anti-HCMV activity of human colostrum in vitro, and we have identified extracellular vesicles as additional antiviral effectors, together with well-known non-specific bioactive and immune components of human colostrum. The investigation of the mechanism of action has indicated that the anti-HCMV activity of EVs is not mediated by a direct inactivation of the viral particle, but rather by the inhibition of the viral attachment to the cell surface. The significant reduction in the antiviral activity of EVs, after shaving experiments, suggests a crucial role of EV surface proteins in inhibiting viral infection. The proteomic analysis of the EV surfaceome revealed the contribution of immune components including antibodies (mainly IgA) and complement component proteins (C9 and C3) to antiviral activity. Moreover, we identified several proteins which have proven antiviral properties against HIV infection but which have scarcely been characterized so far for their activity against HCMV. We found both milk proteins (caseins, whey proteins and milk fat membrane associated proteins) and proteins shared by different tissue cells and biological fluids. The common feature of the identified proteins is that their glycomoieties seem to play a crucial role in the interference mechanism between a virus and the host cells, acting as a point of cell-specific recognition for the virus particle and inhibiting its attachment on the cell surface. Further studies are necessary to explore the involvement of the identified proteins and their glycan chain composition in the protective role of EVs against HCMV.

## Figures and Tables

**Table 1:** Main clinical characteristics of the study group.

Sample no.	Parity *	Breastfeeding after Previous Delivery(ies)	Type of Delivery
1	2002	Yes	CS
2	0000	-	CS
3	0000	-	CS
4	1001	Yes	CS
5	0000	-	S
6	1021	Yes	S
7	0000	-	CS
8	0000	-	CS
9	1001	Yes	CS
10	1001	Yes	CS
11	1001	-	S
12	0000	Yes	CS
13	1001	-	CS

* The numbers indicate full-term births, premature births, abortions and living children. CS: caesarean section; S: spontaneous delivery.

**Table 2:** Anti-human cytomegalovirus (HCMV) activity of the colostra and colostrum-derived EVs.

	Sample no.	EC_50_^a^ (µg/mL) (95% CI ^b^)	EC_90_^c^ (µg/mL) (95% CI)
**Colostra**	1	67.33 (54.24–83.57)	190 (118.5–304.5)
	2	62.58 (50.45–77.63)	215 (133.9–345.3)
	3^d^	153.5 (129.5–182.1)	527.6 (359.1–775.1)
	4	152 (83.46–276.8)	581.8 (160.1–2115)
	5	115.7 (109.3–122.6)	306 (269.7–347.2)
	6	104.3 (84.02–129.5)	652.9 (404.6–1053)
	7	51.35 (28.24–93.38)	647.4 (170.6–2457)
	8	111.8 (90.67–137.9)	323.2 (204.3–511.6)
	9	149.7 (116.9–191.6)	920.1 (545.3–1552)
	10	38.71 (29.82–50.24)	738.3 (405.4–1345)
	11	125.3 (105.9–148.2)	356.8 (245.1–519.4)
	12	140.6 (107.9–198.4)	637.5 (431.6–941.5)
	13	213.9 (186.2–245.7)	413.9 (290.4–590)
**Colostrum-derived EV**	1	35.29 (23.91–52.08)	200.9 (85.08–474.4)
	2	8.84 (5.66–13.81)	120.4 (42.95–337.4)
	3^d^	41.56 (27.44–62.95)	222.2 (87.30–565.7)
	4	42.16 (27.33–65.02)	435.9 (168.3–1129)
	5	55.55 (49.31–62.58)	97.31 (69.92–135.4)
	6	19.68 (14.09–27.49)	479.6 (219.7–1047)
	7	3.98 (1.36–11.63)	844.1 (74.99–9501)
	8	21.96 (13.20–36.53)	277.2 (85.72–896.4)
	9	75.93 (47.92–120.3)	625.1 (201.5–1940)
	10	33.92 (14.31–80.38)	1324 (65.48–26763)
	11	74.21 (62.6–113.3)	493.8 (246.8–988)
	12	41.4 (32.7–64.59)	277.6 (159.7–482.4)
	13	30.92 (22.27–42.94)	371 (187.3–735.1)

^a^ EC_50_: half maximal effective concentration; ^b^ CI: confidence interval; ^c^ EC_90_: 90%-effective concentration; ^d^ HCMV multiplicity of infection (MOI) 0.3 FFU/cell.

**Table 3:** List of the 45 proteins shared between at least two samples out of three (samples 11, 12 and 13) identified by means of LC–MS/MS. The UNIPROT ID, the molecular weight (MW), the isoelectric point (pI) and the biological function are reported for each identified protein.

Uniprot Id	Description	MW	pI	Biological Function
O00300	Tumor necrosis factor receptor superfamily member 11B	45,996	8.66	Signaling pathway
O00391	Sulfhydryl oxidase 1	66,818	9.13	Oxidation-reduction process
O15232	Matrilin-3	52,816	6.25	Calcium ion binding
P00709	Alpha-lactalbumin	16,214	4.83	Lactose biosynthetic process
P01011	Alpha-1-antichymotrypsin	47,621	5.33	Proteolysis
P01024	Complement C3	187,030	6.02	Immune response
P01833	Polymeric immunoglobulin receptor	83,232	5.58	Immune response
P01834	Immunoglobulin kappa constant	11,758	6.11	Immune response
P01876	Immunoglobulin heavy constant alpha 1	37,631	5.99	Immune response
P02647	Apolipoprotein A-I	30,759	5.56	Lipid transport
P02649	Apolipoprotein E	36,132	5.65	Lipid transport
P02652	Apolipoprotein A-II	11,168	6.26	Lipid transport
P02748	Complement component C9	63,133	5.43	Immune response
P02788	Lactotransferrin	78,182	8.50	Immune response
P04114	Apolipoprotein B-100	515,283	6.58	Lipid transport
P04406	Glyceraldehyde-3-phosphate dehydrogenase	36,030	8.57	Glucose metabolic process
P05814	Beta-casein	25,366	5.52	Casein micelle formation
P06396	Gelsolin	85,698	5.90	Cell motility and contraction
P06858	Lipoprotein lipase	53,129	8.37	Lipid metabolic process
P07498	Kappa-casein	20,293	8.97	Casein micelle formation
P07602	Prosaposin	58,112	5.06	Lipid metabolic process
P07996	Thrombospondin-1	129,300	4.71	Adhesion
P08571	Monocyte differentiation antigen CD14	40,051	5.84	Immune response
P10451	Osteopontin	35,422	4.37	Adhesion
P10909	Clusterin	52,494	5.88	Protein folding/transport
P15291	Beta-1,4-galactosyltransferase 1	43,920	8.88	Carbohydrate metabolic process
P19835	Bile salt-activated lipase	79,272	5.13	Lipid metabolic process
P22897	Macrophage mannose receptor 1	165,905	6.11	Endocytosis
P23280	Carbonic anhydrase 6	35,366	6.51	Carbon metabolic process
P23284	Peptidyl-prolyl cis-trans isomerase B	23,728	9.42	Protein folding/transport
P24821	Tenascin	240,853	4.79	Adhesion
P47710	Alpha-S1-casein	21,671	5.32	Calcium ion binding
P47989	Xanthine dehydrogenase/oxidase	146,330	7.86	Oxidation-reduction process
P49327	Fatty acid synthase	273,254	6.01	Oxidation-reduction process
P58499	Protein FAM3B	25,981	8.97	Insulin secretion
P60709	Actin, cytoplasmic 1	41,710	5.29	Cell motility and contraction
Q02809	Procollagen-lysine,2-oxoglutarate 5-dioxygenase 1	83,550	6.46	Oxidation-reduction process
Q08431	Lactadherin	43,105	8.47	Adhesion
Q13410	Butyrophilin subfamily 1 member A1	58,923	5.38	Milk-fat droplet release / immune response
Q14697	Neutral alpha-glucosidase AB	106,873	5.74	Carbohydrate metabolic process
Q6WN34	Chordin-like protein 2	49,643	6.75	Signaling pathway
Q96DA0	Zymogen granule protein 16 homolog B	22,725	6.74	Protein folding/transport
Q96S86	Hyaluronan and proteoglycan link protein 3	40,868	6.07	Adhesion
Q99102	Isoform 10 of Mucin-4	225,293	5.85	Adhesion
Q9H173	Nucleotide exchange factor SIL1	52,052	5.27	Protein folding/transport

## Supplementary Materials

The following are available online at https://www.mdpi.com/2076-2607/8/7/1087/s1. Table S1. List of the proteins identified by means of LC–MS/MS in at least two samples out of three. The protein score and the sequence of the detected peptides are reported for each protein. Figure S1. Cell viability assay. Evaluation of the effect of three EV preparations (EV1, EV2 and EV3) on HFF-1 viability at 5 days. The HFF-1 cells were treated under the same conditions as the antiviral assays and processed by means of an MTS assay. Data are reported as the percentages of viable cells in comparison with the controls, as determined by means of the MTS assay for 1000, 500, 250 and 125 µg/mL concentrations. Three independent experiments were performed.
