# Supplementary material for: Extracellular Vesicles in Human Preterm Colostrum Inhibit Infection by Human Cytomegalovirus In Vitro
Source: Microorganisms. 2020 Jul 21;8(7):1087. doi: 10.3390/microorganisms8071087 (PMC7409124; doi:10.3390/microorganisms8071087)
Supplement: Supplementary file 1 [file microorganisms-08-01087-s001.pdf]

Table S1. List of the proteins identified by LC-MS/MS in at least two samples out of three. The protein score and the sequence of the detected peptides are reported for each protein.

				sample 11			sample 12			sample 13		
**UNIPROT ID**	**Description**	**MW**	**pI**	**Score**	**N° peptides**	peptide sequences	Score	N° peptides	peptide sequences	Score	N° peptides	peptide sequences
O00300	Tumor necrosis factor receptor superfamily member 11B	45996	8.66	116	3	TVTQSLK<br>VGAEDIEK<br>SLMESLPGKK	92	3	TVTQSLK<br>VGAEDIEK<br>SLMESLPGKK			n.i.
O00391	Sulfhydryl oxidase 1	66818	8.85	129	1	DVQNVAAAPELAMGALELESR	257	7	GAVLGSR<br>EGAVLAK<br>VPVLMESR<br>VLNTEANVVR<br>AAPGQEPPEHMAELQR<br>AHFSPSNIILDFPAAGSAAR<br>RDVQNVAAAPELAMGALELESR			n.i.
O15232	Matrilin-3	52816	6.25	194	3	EPSSNIPK<br>RPSPAAPDGAPASGTSEPGR<br>MMASEPLEEHVFYVETYGVIEK	346	6	EPSSNIPK<br>IEFQLQAYTDK<br>IIDTLDIGPADTR<br>AQASGIELYAVGVDR<br>VAIIVTDGRPQDQVNEVAAR<br>MMASEPLEEHVFYVETYGVIEK			n.i.
P00709	Alpha-lactalbumin	16214	4.83	55	2	SSQVPQSR<br>GIDYWLAHK	211	3	SSQVPQSR<br>CELSQLLK<br>GIDYWLAHK			n.i.
P01011	Alpha-1-antichymotrypsin	47621	5.33	373	4	NLAVSQVVHK<br>ITLLSALVETR<br>AVLDVFEEGTEASAATAVK<br>DYNLNDILLQLGIEEAFTSK	180	2	EQLSLLDR<br>AVLDVFEEGTEASAATAVK			n.i.
P01024	Complement C3	187030	6.02	1368	9	QPSSAFAAFVK<br>DFDFVPPVVR<br>ENEGFTVTAEGK<br>EVVADSVWVDVK<br>SEETKENEGFTVTAEGK<br>DAPDHQELNLDVSLQLPSR<br>ILLQGTPVAQMTEDAVDAER<br>VPVAVQGEDTVQSLTQGDGVAK<br>EGVQKEDIPPADLSDQVPDTESETR	1509	19	LVLSSEK<br>SVQLTEK<br>NEQVEIR<br>TGLQEVEVK<br>QPSSAFAAFVK<br>DFDFVPPVVR<br>APSTWLTAYVVK<br>KQELSEAEQATR<br>SSLSVPYVIVPLK<br>AGDFLEANYMNLQR<br>SNLDEDIIAEENIVSR<br>SEETKENEGFTVTAEGK<br>TELRPGETLNVNFLLR<br>LSINTHPSQKPLSITVR<br>DAPDHQELNLDVSLQLPSR<br>ILLQGTPVAQMTEDAVDAER<br>SLYVSATVILHSGSDMVQAER<br>YYGGGYGSTQATFMVFQALAQYQK<br>EGVQKEDIPPADLSDQVPDTESETR	412	3	SEETKENEGFTVTAEGK<br>ILLQGTPVAQMTEDAVDAERLK<br>EGVQKEDIPPADLSDQVPDTESETR
P01833	Polymeric immunoglobulin receptor	83232	5.58	1780	14	APAFEGR<br>VLDSGFR<br>VYTVDLGR<br>ILLNPQDK<br>IIEGEPNLK<br>LVSLTLNLVTR<br>DGSFSVVITGLR<br>DGSFSVVITGLRK<br>QGHFYGETAAVYVAVEER<br>FLCRQSSGENCDVVVNTLGK<br>ILLNPQDKDGSFSVVITGLR<br>GLSFDVSLEVSQGPGLLNDTK<br>NADLQVLKPEPELVYEDLR<br>ILLNPQDKDGSFSVVITGLRK	2063	13	APAFEGR<br>VLDSGFR<br>VYTVDLGR<br>ILLNPQDK<br>IIEGEPNLK<br>LVSLTLNLVTR<br>DGSFSVVITGLR<br>QGHFYGETAAVYVAVEER<br>LDIQGTGQLLFSVVINQLR<br>ILLNPQDKDGSFSVVITGLR<br>NADLQVLKPEPELVYEDLR<br>KNADLQVLKPEPELVYEDLR<br>IRLDIQGTGQLLFSVVINQLR	599	5	VLDSGFR<br>VLDSGFREIENK<br>DVSLAKADAAPDEK<br>ADAAPDEKVLDSGFR<br>ADAAPDEKVLDSGFREIENK
P01834	Immunoglobulin kappa constant	11758	6.11	602	3	DSTYSLSSTLTLSK<br>TVAAPSVFIFPPSDEQLK<br>VDNALQSGNSQESVTEQDSK	218	3	DSTYSLSSTLTLSK<br>TVAAPSVFIFPPSDEQLK<br>VDNALQSGNSQESVTEQDSK			n.i.
P01876	Immunoglobulin heavy constant alpha 1	37631	6.08	317	3	TPLTATLSK<br>DASGVTFTWTPSSGK<br>QEPSQGTTTFAVTSILR	393	3	SAVQGPPER<br>DASGVTFTWTPSSGK<br>QEPSQGTTTFAVTSILR			n.i.
P02647	Apolipoprotein A-I	30759	5.56	543	6	DLATVYVDVLK<br>VSFLSALEEYTK<br>DYVSQFEGSALGK<br>DSGRDYVSQFEGSALGK<br>EQLGPVTQEFWDNLEK	746	15	AHVDALR<br>AELQEGAR<br>LHELQEK<br>AKPALEDLR<br>LSPLGEEMR			n.i.

						LREQLGPVTQEFWDNLEKETEGLR			LEALKENGGAR<br>ATEHLSTLSEK<br>DLATVYVDVLK<br>VQPYLDDFQK<br>THLAPYSDELR<br>VSFLSALEEYTK<br>DYVSQFEGSALGK<br>LLDNWDSVTSTFSK<br>EQLGPVTQEFWDNLEK<br>LREQLGPVTQEFWDNLEK			
P02649	Apolipoprotein E	36132	5.65	397	3	VQAAVGTSAAPVPSDNH<br>SELEEQLTPVAEETR<br>WVQTLSEQVQEELLSSQVTQELR	322	5	ALMDETMK<br>SWFEPLVEDMQR<br>VQAAVGTSAAPVPSDNH<br>SELEEQLTPVAEETR<br>WVQTLSEQVQEELLSSQVTQELR			n.i.
P02652	Apolipoprotein A-II	11168	6.26	213	4	SPELQAEAK<br>EQLTPLIKK<br>AGTELVNFLSYFVELGTQPATQ<br>KAGTELVNFLSYFVELGTQPATQ	131	4	SPELQAEAK<br>SKEQLTPLIK<br>VKSPELQAEAK<br>AGTELVNFLSYFVELGTQPATQ	47	1	GKVKVGVNGFGR
P02748	Complement component C9	63133	5.43	112	1	GTVIDVTDFVNWASSINDAPVLISQK	68	2	TSNFNAAISLK<br>LSPIYNLVPVK			n.i.
P02788-2	Lactotransferrin	78182	8.50	486	5	DGAGDVAFIR<br>SDTSLTWNSVK<br>YLGPQYVAGITNLKK<br>GEADAMSLDGGYVYTAGK<br>DVTVLQNTDGNNNEAWAK	2491	23	DSAIGFSR<br>VPSHAVVAR<br>EDAIWNLLR<br>THYYAVAVVK<br>FQLFGSPSGQK<br>SDTSLTWNSVK<br>THYYAVAVVKK<br>GGSFQLNELQGLK<br>LRPVAAEVYGTER<br>DLLFKDSAIGFSR<br>KGGSFQLNELQGLK<br>SVNGKEDAIWNLLR<br>YLGPQYVAGITNLKK<br>GEADAMSLDGGYVYTAGK<br>FQLFGSPSGQKDLLFK<br>LRPVAAEVYGTERQPR<br>ADAVTLDGGFIYEAGLAPYK<br>IDSGLYLGSGYFTAIQNLR<br>IDSGLYLGSGYFTAIQNLRK<br>VPPRIDSGLYLGSGYFTAIQNLR<br>FQLFGSPSGQKDLLFKDSAIGFSR<br>VPPRIDSGLYLGSGYFTAIQNLRK<br>ADAVTLDGGFIYEAGLAPYK<br>LRPVAAEVYGTER			n.i.
P04114	Apolipoprotein B-100	515283	6.58	214	3	FPEVDVLTK<br>SVSDGIAALDLNAVANK<br>VPSYTLILPSLELPVLHVPR	883	14	FVTQAEGAK<br>QIDDIDVR<br>FPEVDVLTK<br>TEVIPPLIENR<br>AASGTTGTYQEWK<br>AVSMPSFSILGSDVR<br>TSSFALNLPTLPEVK<br>ALYWVNGQVPDGVSK<br>SVSDGIAALDLNAVANK<br>NIQEYLSILTDPDGK<br>VNWEEEAASGLLTSLK<br>VPSYTLILPSLELPVLHVPR<br>VNWEEEAASGLLTSLKDNVPK<br>IADFELPTIIVPEQTIEIPSIK			n.i.
P04406	Glyceraldehyde-3-phosphate dehydrogenase	36030	8.57	171	2	VIISAPSADAPMFVMGVNHEK<br>VIHDNFGIVEGLMTTVHAITATQK	300	5	VGVNGFGR<br>GALQNIIPASTGAAK<br>LVINGNPITIFQER<br>VIISAPSADAPMFVMGVNHEK<br>VIHDNFGIVEGLMTTVHAITATQK	47	2	GKVKVGVNGFGR<br>DGRGALQNIIPASTGAAK
P05814	Beta-casein	25366	5.52	2447	6	SPTIPFFDPQIPK<br>VLPIPQQVVPYPQR<br>ETIESLSSSEESITEYK<br>AVPVQALLLNQELLLNPTHQIYPVTQPLAPVHNPISVLTDLENLHLPLPLLQPLMQQVPQPIPQTLALPPQPLWSVPQPK<br>VKHEDQQQGEDEHQDKIYPSFQPQPLIYPFVEPIPYGFLPQNILPLAQPAVVLPVPQPEIMEVPK	4325	10	AKDTVYTK<br>SPTIPFFDPQIPK<br>VLPIPQQVVPYPQR<br>ETIESLSSSEESITEYK<br>VKHEDQQQGEDEHQDK<br>VMPVLKSPTIPFFDPQIPK<br>AVPVQALLLNQELLLNPTHQIYPVTQPLAPVHNPISVLTDLENLHLPLPLLQPLMQQVPQPIPQTLALPPQPLWSVPQPK<br>IYPSFQPQPLIYPFVEPIPYGFLPQNILPLAQPAVVLPVPQPEIMEVPK<br>VKHEDQQQGEDEHQDKIYPSFQPQPLIYPFVEPIPYGFLPQNILPLAQPAVVLPVPQPEIMEVPK	2660	13	GRVMPVLK<br>AKDTVYTK<br>DTVYTKGR<br>SPTIPFFDPQIPK<br>VLPIPQQVVPYPQR<br>ETIESLSSSEESITEYK<br>VMPVLKSPTIPFFDPQIPK<br>GRVMPVLKSPTIPFFDPQIPK<br>AVPVQALLLNQELLLNPTHQIYPVTQPLAPVHNPISV<br>LTDLENLHLPLPLLQPLMQQVPQPIPQTLALPPQPLWSVPQPK

												IYPSFQPQPLIYPFVEPIPYGFL<br>PQNILPLAQPAVVLPVPQPEI<br>MEVPK<br>SPTIPFFDPQIPKLTDLENLHL<br>PLPLLQPLMQQVPQPIPQTLA<br>LPPQPLWSVPQPK<br>VKHEDQQQGEDEHQDKIYPS<br>FQPQPLIYPFVEPIPYGFLPQNI<br>LPLAQPAVVLPVPQPEIMEVP<br>K
P06396	Gelsolin	85698	5.90	107	2	GGVASGFK<br>TGAQELLR	331	6	YIETDPANR<br>HVVPNEVVVQR<br>EVQGFESATFLGYFK<br>EPAHLMSLFGGKPMIIYK<br>DPDQTDGLGLSYLSSHIANVE<br>R<br>VPFDAATLHTSTAMAAQHG<br>MDDDGTGQK			n.i.
P06858	Lipoprotein lipase	53129	8.4	534	7	APAVFVK<br>LVGQDVAR<br>DFIDIESK<br>GLGDVDQLVK<br>EPDSNVIVVDWLSR<br>SIHLFIDSLLNEENPSK<br>ITGLDPAGPNFEYAEAPSR	648	11	APAVFVK<br>LVGQDVAR<br>DFIDIESK<br>GLGDVDQLVK<br>AQEHYPVSAGYTK<br>EPDSNVIVVDWLSR<br>LSPDDADFVDVLHTFTR<br>SIHLFIDSLLNEENPSK<br>ITGLDPAGPNFEYAEAPSR<br>TFMVIHGWTVTGMYESWVP<br>K<br>FINWMEEEFNYPLDNVHLLG<br>YSLGAHAAGIAGSLTNK	277	6	DFIDIESK<br>GLGDVDQLVK<br>LSPDDADFVDVLHTFTR<br>SIHLFIDSLLNEENPSK<br>ITGLDPAGPNFEYAEAPSRLSP<br>DDADFVDVLHTFTR<br>FINWMEEEFNYPLDNVHLLG<br>YSLGAHAAGIAGSLTNK
P07498	Kappa-casein	20293	8.97	1417	5	RPAIAINNPYVPR<br>QYLPNSHPPTVVR<br>RPNLHPSFIAIPPK<br>RPNLHPSFIAIPPKK<br>TYYANPAVVRPHAQIPQR	1626	8	RPAIAINNPYVPR<br>QYLPNSHPPTVVR<br>RPNLHPSFIAIPPK<br>RPNLHPSFIAIPPKK<br>TYYANPAVVRPHAQIPQR<br>SFLLVVNALALTLPFLAVEVQ<br>NQK<br>MKSFLLVVNALALTLPFLAVE<br>VQNQK<br>TAPYVPMYYVPNSYPYYGTN<br>LYQR	3509	10	RPAIAINNPYVPR<br>QYLPNSHPPTVVR<br>RPNLHPSFIAIPPK<br>RPNLHPSFIAIPPKK<br>TYYANPAVVRPHAQIPQR<br>RPNLHPSFIAIPPKKIQDK<br>TAPYVPMYYVPNSYPYYGTN<br>LYQR<br>QYLPNSHPPTVVRRPNLHPSF<br>IAIPPK<br>RPAIAINNPYVPRTYYANPAV<br>VRPHAQIPQR<br>TYYANPAVVRPHAQIPQRQY<br>LPNSHPPTVVR
P07602	Prosaposin	58112	5.06	305	4	LPALTVHVTQPK<br>NVIPALELVEPIK<br>NVIPALELVEPIKK<br>IPELDMTEVVAPFMANIPLL<br>LYPQDGPR	92	1	NVIPALELVEPIKK			n.i.
P07996	Thrombospondin-1	129300	4.71	362	6	DLASIAR<br>GPDPSSPAFR<br>IEDANLIPPVPDDK<br>GGVNDNFQGVLQNVR<br>MENAELDVPIQSVFTR<br>IEDANLIPPVPDDKFQDLVD<br>AVR	207	5	DLASIAR<br>SITLFVQEDR<br>AGTLDLSLTVQGK<br>GGVNDNFQGVLQNVR<br>IEDANLIPPVPDDKFQDLVDA<br>VR			n.i.
P08571	Monocyte differentiation antigen CD14	40051	5.84	282	5	ELTLEDLK<br>LKELTLEDLK<br>LTVGAAQVPAQLLVGALR<br>RLTVGAAQVPAQLLVGALR<br>ITGTMPPLPLEATGLALSSLR	609	10	QYADTVK<br>ELTLEDLK<br>FPAIQNLALR<br>LKELTLEDLK<br>STLSVGVSGTLVLLQGAR<br>LTVGAAQVPAQLLVGALR<br>SWLAELQQWLKPGLK<br>RLTVGAAQVPAQLLVGALR<br>AFPALTSLDLSDNPGLGER<br>ITGTMPPLPLEATGLALSSLR			n.i.
P10451	Osteopontin	35442	4.37	860	7	GDSVVYGLR<br>QNLLAPQTLPSK<br>YPDAVATWLNPDPSQK<br>AIPVAQDLNAPSDWDSR<br>GKDSYETSQLDDQSAETHSH<br>K<br>QLYNKYPDAVATWLNPDPS<br>QK<br>QETLPSK	1303	8	GDSVVYGLR<br>QNLLAPQTLPSK<br>ISHELDSASSEVN<br>YPDAVATWLNPDPSQK<br>AIPVAQDLNAPSDWDSR<br>QLYNKYPDAVATWLNPDPS<br>QK<br>RPDIQYPDATDEDITSHMESE<br>ELNGAYK<br>FRRPDIQYPDATDEDITSHME<br>SEELNGAYK			n.i.
P10909	Clusterin	52494	5.88	4229	16	SGSGLVGR<br>FMETVAEK<br>EIQNAVNGVK<br>IDSLLENDR<br>TLLSNLEEAK<br>TLLSNLEEAKK<br>ELDESLQVAER<br>KTLLSNLEEAKK<br>ASSIIDELFQDR<br>QQTHMLDVMQDHFSR<br>LFDSDPITVTVPVEVSR<br>EPQDTYHYLPFSLPHR<br>LFDSDPITVTVPVEVSRK<br>VTTVASHTSDSDVPSGVTEV<br>VVK<br>IDSLLENDRQQTHMLDVMQ<br>DHFSR<br>MQDHFSR	2096	15	SGSGLVGR<br>FMETVAEK<br>EIQNAVNGVK<br>IDSLLENDR<br>TLLSNLEEAK<br>TLLSNLEEAKK<br>KTLLSNLEEAK<br>ELDESLQVAER<br>KTLLSNLEEAKK<br>ASSIIDELFQDR<br>RELDESLQVAER<br>YVNKEIQNAVNGVK<br>QQTHMLDVMQDHFSR<br>LFDSDPITVTVPVEVSR<br>VTTVASHTSDSDVPSGVTEVV<br>VK	1931	20	FMETVAEK<br>EIQNAVNGVK<br>TLLSNLEEAK<br>TLIEKTNEER<br>ELDESLQVAER<br>NPKFMETVAEK<br>TLIEKTNEERK<br>ASSIIDELFQDR<br>YVNKEIQNAVNGVK<br>ELDESLQVAERLTR<br>FMETVAEKALQEYR<br>LFDSDPITVTVPVEVSR<br>TNEERKTLLSNLEEAK<br>YVNKEIQNAVNGVKQIK<br>EPQDTYHYLPFSLPHR<br>LFDSDPITVTVPVEVSRK<br>VTTVASHTSDSDVPSGVTEVV<br>VK

												LFDSDPITVTVPVEVSRKNPK<br>ASSIIDELFQDRFFTREPQDTY<br>HYLPFSLPHR<br>VTTVASHTSDSDVPSGVTEVV<br>VKLFDSDPITVTVPVEVSR
P15291	Beta-1,4-galactosyltransferase 1	43920	8.88	707	6	HISVAMDK<br>LLNVGFQEALK<br>GMSISRPNAVVGR<br>YPLYTQITVDIGTPS<br>ETMLSDGLNSLTYQVLDVQ<br>R<br>LPQLVGVSTPLQGGSNSAA<br>AIGQSSGELR	372	5	HISVAMDK<br>VAIIIPFR<br>LLNVGFQEALK<br>ETMLSDGLNSLTYQVLDVQR<br>LPQLVGVSTPLQGGSNSAAAI<br>GQSSGELR	274	2	LPQLVGVSTPLQGGSNSAAAI<br>GQSSGELR<br>DLSRLPQLVGVSTPLQGGSNS<br>AAAIGQSSGELR
P19835	Bile salt-activated lipase	79272	5.13	1782	17	GIPFAAPTK<br>EAQMPAVIR<br>VTEEDFYK<br>LVSEFTITK<br>DQHMAIAWVK<br>LGLLGDSVDIFK<br>LGAVYTEGGFVEGVNK<br>AISQSGVALSPWVIQK<br>VGPLGFLSTGDANLPGNYG<br>LR<br>TTFDVYTESWAQDPSQENK<br>TTFDVYTESWAQDPSQENK<br>K<br>TVVDFETDVLFLVPTEIALA<br>QHR<br>KTVVDFETDVLFLVPTEIALA<br>QHR<br>CLQATITQDSTYGDEDCLYL<br>NIWVPQGR<br>WVGADHADDIQYVFGKPF<br>ATPTGYRPQDR<br>TGDPNMGDSAVPTHWEPYT<br>TENSGYLEITK<br>TGDPNMGDSAVPTHWEPYT<br>TENSGYLEITKK	6019	33	MGSSSMK<br>ALTLAYK<br>NPLFWAK<br>GIPFAAPTK<br>EAQMPAVIR<br>VTEEDFYK<br>LVSEFTITK<br>KVTEEDFYK<br>EAQMPAVIRF<br>DQHMAIAWVK<br>LGLLGDSVDIFK<br>GNVIVVTFNYR<br>AMIAYWTNFAK<br>TYAYLFSHPSR<br>KLGLLGDSVDIFK<br>LGAVYTEGGFVEGVNK<br>AISQSGVALSPWVIQK<br>LGAVYTEGGFVEGVNKK<br>ALENPQPHPGWQGTLK<br>RAISQSGVALSPWVIQK<br>VGPLGFLSTGDANLPGNYGL<br>R<br>LGLLGDSVDIFKGIPFAAPTK<br>TTFDVYTESWAQDPSQENKK<br>TVVDFETDVLFLVPTEIALAQ<br>HR<br>KTVVDFETDVLFLVPTEIALA<br>QHR<br>WVGADHADDIQYVFGKPFA<br>TPTGYRPQDR<br>TGDPNMGDSAVPTHWEPYT<br>TENSGYLEITK<br>NIAAFGGDPNNITLFGESAGG<br>ASVSLQTLSPYNK<br>TGDPNMGDSAVPTHWEPYT<br>TENSGYLEITKK<br>RNIAAFGGDPNNITLFGESAG<br>GASVSLQTLSPYNK<br>DLPVMIWIYGGAFLMGSGHG<br>ANFLNNYLYDGEEIATR<br>VPLAGLEYPMLHYVGFVPVID<br>GDFIPADPINLYANAADIDYI<br>AGTNNMDGHIFASIDMPAIN<br>K	1409	15	NPLFWAK<br>GIPFAAPTK<br>LVSEFTITK<br>LGLLGDSVDIFK<br>ALENPQPHPGWQGTLK<br>VTEEDFYKLVSEFTITK<br>LGLLGDSVDIFKGIPFAAPTK<br>TTFDVYTESWAQDPSQENK<br>TTFDVYTESWAQDPSQENKK<br>TTFDVYTESWAQDPSQENKK<br>K<br>AISQSGVALSPWVIQKNPLFW<br>AK<br>TVVDFETDVLFLVPTEIALAQ<br>HR<br>KTVVDFETDVLFLVPTEIALA<br>QHR<br>KKTVVDFETDVLFLVPTEIAL<br>AQHR<br>WVGADHADDIQYVFGKPFA<br>TPTGYRPQDR
P22897	Macrophage mannose receptor 1	165905	6.11	122	2	YTNWAADEPK<br>DSTFSAWTGLNDVNSEHTFL<br>WTDGR	3559	37	GSGLWSR<br>SSYSLMR<br>FAWMDGSK<br>KTWFESR<br>GVHYTNWGK<br>LITASGSYHK<br>EEQQTIWR<br>DYQYYFSK<br>EGWNFYSNK<br>IFGFMEEER<br>ALGGDLASINNK<br>YTNWAADEPK<br>SQGPEIVEVEK<br>TGIAGGLWDVLK<br>EQAFLTYHMK<br>IFGFMEEERK<br>GEDLFFNYGNR<br>FTHWNSDMPGR<br>YFWTGLSDIQTK<br>QFLIYNEDHKR<br>GTFQWTIEEEVR<br>WVSESQIMSVAFK<br>NFGDLVSIQSESEK<br>NFGDLVSIQSESEKK<br>RNFGDLVSIQSESEK<br>RNFGDLVSIQSESEKK<br>NDAQSAYFIGLLISLDK<br>YEQAFLTSFVGLRPEK<br>SDPSLTNPPATIQTDGFVK<br>NDAQSAYFIGLLISLDKK<br>IQMYFEWSDGTPVTFTK<br>MGSSLVSIESAAESSFLSYR<br>ALGGDLASINNKEEQQTIWR	n.i.		

									YVNRNDAQSAYFIGLLISLDK<br>K<br>FEGSESLWNKDPLTSVSYQIN<br>SK<br>DSTFSAWTGLNDVNSEHTFL<br>WTDGR<br>LHNSLIASILDPYSNAFAWLQ<br>METSNER			
P23280	Carbonic anhydrase 6	35366	6.51	376	4	LENSLLDHR<br>TTLTGLDVQDMLPR<br>NYPENTYYSNFISHLANIK<br>VVESNFPNQEYTLGSEFQFY<br>LHK			n.i.	65	1	VVESNFPNQEYTLGSEFQFYL<br>HKIEEILDYLR
P23284	Peptidyl-prolyl cis-trans isomerase B	23728	9.42	63	1	TVDNFVALATGEK	106	3	IGDEDVGR<br>VLEGMEVVR<br>TVDNFVALATGEK			n.i.
P24821	Tenascin	24085	4.79	7173	50	SSTITAK<br>FSVGDAK<br>AVDIPGLK<br>STDLPGLK<br>SFSTFDK<br>VSIYGVIR<br>AYAAGFGDR<br>TRDITGLR<br>IQALNGPLR<br>FTTDLDSPR<br>LDAPSQIEVK<br>AATHYTITIR<br>TPVLSAEASTAK<br>ITAQGQYELR<br>ETFTTGLDAPR<br>DHGETAFAVYDK<br>GHEHSIQFAEMK<br>AVDIPGLEAATPYR<br>EEFWLGLDNLNK<br>QSEPLEITLLAPER<br>REEFWLGLDNLNK<br>EATEYEIELYGISK<br>YAPISGGDHAEVDVPK<br>KQSEPLEITLLAPER<br>GLEPGQEYNVLLTAEK<br>ESNPATINAATELDTPK<br>LSWTADEGVFDNFVLK<br>VEGYSGTAGDSMAYHNGR<br>ASVTGYLLVYESVDGTVK<br>DLQVSETAETSLTLLWK<br>TTLTGLRPGTEYGIGVSAVK<br>DHGETAFAVYDKFSVGDAK<br>WQPAIATVDSYVISYTGEK<br>EDKESNPATINAATELDTPK<br>DLTATEVQSETALLTWRPPR<br>TTLTGLRPGTEYGIGVSAVK<br>EDK<br>ASTEQAPELENLTVTEVGW<br>DGLR<br>TVSGNTVEYALTDLEPATEY<br>TLR<br>TAHISGLPPSTDFIVYLSGLA<br>PSIR<br>EVIVGPDTTSYSLADLSPSTH<br>YTAK<br>WQPAIATVDSYVISYTGEKV<br>PEITR<br>TISATATTEAEPEVDNLLVSD<br>ATPDGFR<br>DVTDTTALITWFKPLAEIDGI<br>ELTYGIK<br>TKTISATATTEAEPEVDNLL<br>VSDATPDGFR<br>DLAPPSEPSESFQEHTVDGE<br>NQIVFTHR<br>TTIDLTEDENQYSIGNLKPDT<br>EYEVSLISR<br>GHSTRPLAVEVVTEDLPQLG<br>DLAVSEVGWDGLR<br>ESNPATINAATELDTPKDLQ<br>VSETAETSLTLLWK<br>EDKESNPATINAATELDTPK<br>DLQVSETAETSLTLLWK<br>TTLTGLRPGTEYGIGVSAVK<br>EDKESNPATINAATELDTPK	4702	53	SSTITAK<br>FSVGDAK<br>AVDIPGLK<br>SFSTFDK<br>LRPSNFR<br>SMEIPGLR<br>VSIYGVIR<br>AYAAGFGDR<br>IQALNGPLR<br>FTTDLDSPR<br>AYAAGFGDRR<br>LDAPSQIEVK<br>AATHYTITIR<br>TPVLSAEASTAK<br>ITAQGQYELR<br>ETFTTGLDAPR<br>VATYLPAPEGLK<br>DHGETAFAVYDK<br>GHEHSIQFAEMK<br>AVDIPGLEAATPYR<br>AGTPYTVTLHGEVR<br>VSQTDNSITLEWR<br>QSEPLEITLLAPER<br>EATEYEIELYGISK<br>YAPISGGDHAEVDVPK<br>KQSEPLEITLLAPER<br>GLEPGQEYNVLLTAEK<br>ESNPATINAATELDTPK<br>LSWTADEGVFDNFVLK<br>AATPYTVSIYGVIQGYR<br>VEGYSGTAGDSMAYHNGR<br>ASVTGYLLVYESVDGTVK<br>DLQVSETAETSLTLLWK<br>QTGLAPGQEYEISLHIVK<br>YGDNNHSQGVNWFHWK<br>TTLTGLRPGTEYGIGVSAVK<br>LKVEGYSGTAGDSMAYHNG<br>R<br>WQPAIATVDSYVISYTGEK<br>EDKESNPATINAATELDTPK<br>DLTATEVQSETALLTWRPPR<br>TTLTGLRPGTEYGIGVSAVKE<br>DK<br>VTEYLVVYTPTHEGGLEMQF<br>R<br>VPGDQTSTIIQELEPGVEYFIR<br>ASTEQAPELENLTVTEVGWD<br>GLR<br>TVSGNTVEYALTDLEPATEYT<br>LR<br>TVSGNTVEYALTDLEPATEYT<br>LR<br>TAHISGLPPSTDFIVYLSGLAP<br>SIR<br>EVIVGPDTTSYSLADLSPSTHY<br>TAK<br>WQPAIATVDSYVISYTGEKVP<br>EITR<br>TISATATTEAEPEVDNLLVSD<br>ATPDGFR<br>DVTDTTALITWFKPLAEIDGIE<br>LTYGIK<br>DLAPPSEPSESFQEHTVDGEN<br>QIVFTHR<br>GHSTRPLAVEVVTEDLPQLG<br>DLAVSEVGWDGLR	253	4	GLEPGQEYNVLLTAEK<br>VDLRDHGETAFAVYDKFSVG<br>DAK<br>IKYAPISGGDHAEVDVPKSQQ<br>ATTK<br>WQPAIATVDSYVISYTGEKVP<br>EITR
P47710	Alpha-S1-casein	21671	5.32	2154	9	NNVMLQW<br>LPLRYPER<br>QTDEIKDTR<br>EEYMNGMNR<br>EKQTDEIKDTR<br>MESSISSSSEEMSLSK<br>LQNPSESSEPIPLESR<br>LNEYNQLQLQAAHAQEQIR<br>LNEYNQLQLQAAHAQEQIR<br>R	4584	12	NNVMLQW<br>EKQTDEIK<br>LPLRYPER<br>QTDEIKDTR<br>EEYMNGMNR<br>EKQTDEIKDTR<br>MESSISSSSEEMSLSK<br>LQNPSESSEPIPLESR<br>LNEYNQLQLQAAHAQEQIR<br>LNEYNQLQLQAAHAQEQIRR	285	5	NNVMLQW<br>LPLRYPER<br>EEYMNGMNR<br>LQNPSESSEPIPLESR<br>LQNPSESSEPIPLESREEYMNG<br>MNR

									MNENSHVQVPFQQLNQLAA<br>YPYAVWYYPQIMQYVPFPPFS<br>DISNPTAHENYEK<br>RMNENSHVQVPFQQLNQLA<br>AYPYAVWYYPQIMQYVPFPP<br>FSDISNPTAHENYEK			
P47989	Xanthine dehydrogenase/oxidase	146330	7.86	178	4	DPPADVQLFQEVPK<br>ITYEELPAIITIEDAIK<br>TLLSPEEILLSIEIPYSR<br>SVASVGGNIITASPISDLNPV<br>FMASGAK	1232	22	LGLSGTK<br>MGGGFGGK<br>MVQVASR<br>GVLEQLR<br>TLVDAVAK<br>MLGVPANR<br>LDSPATPEK<br>LGQENLEDK<br>EGEYFSAFK<br>DEDMLITGGR<br>VTWIQASTLK<br>NNSFYGPELK<br>EGDLTHFNQK<br>IPAFGSIPIEFR<br>TADKLVFFVNGR<br>STVVSTAVALAAYK<br>LVVGNTEIGIEMK<br>NADPETTLLAYLR<br>LDPTFASATLLFQK<br>DPPADVQLFQEVPK<br>TVQMDHTFFPGYR<br>SVASVGGNIITASPISDLNPVF<br>MASGAK	44	2	IEKGDLKK<br>SVASVGGNIITASPISDLNPVF<br>MASGAK
P49327	Fatty acid synthase	273254	6.01	64	1	GVDLVLNSLAEEK	516	9	LLEQGLR<br>VFTTVGSAEK<br>VTAIHIDPATHR<br>VVVQVLAEEPEAVLK<br>LPEDPLLSGLLDSPALK<br>VTVAGGVHISGLHTESAPR<br>LQLNGNLQLELAQVLAQERP<br>K<br>LHLSGIDANPNALFPPVEFPA<br>PR<br>ALGLGVEQLPVVFEDVVLHQ<br>ATILPK	86	2	TPEAVQK<br>GGNVGINSFGFGGSNVHIILR<br>PNTQPPPAPAPHATLPR
P58499	Protein FAM3B	25981	8.97	64	2	NAIEALGSK<br>GLELPSEIQR	20	1	LLSGGGR			n.i.
P60709	Actin, cytoplasmic 1	41710	5.29	210	2	VAPEEHPVLLTEAPLNPK<br>TTGIVMDSGDGVTHTVPIYE<br>GYALPHAILR	145	8	IIAPPER<br>EITALAPSTMK<br>HQGVMVGMGQK<br>TTGIVMDSGDGVTHTVPIYEG<br>YALPHAILR<br>IIAPPER<br>EITALAPSTMK<br>HQGVMVGMGQK<br>YPIEHGIITNWDDMEK	56	1	VAPEEHPVLLTEAPLNPK
Q02809	Procollagen-lysine,2-oxoglutarate 5-dioxygenase 1	83550	6.46	184	2	NLAYDTLPVLIHGNGPTK<br>IQGGYENVPTIDIHMNQIGF<br>ER	92	1	IFQNLDGALDEVVLK			n.i.
Q08431	Lactadherin	43105	8.47	195	2	NAVHVNLFETPVEAQYVR<br>AGMVNAWTPSSNDDNPWI<br>QVNLLR	66	1	NAVHVNLFETPVEAQYVR			n.i.
Q13410	Butyrophilin subfamily 1 member A1	58923	5.38	807	8	FPSTSESR<br>ATLVQDGIAK<br>TPLPLAGPPR<br>GEKFPSTSESR<br>EQEAEQMPEYR<br>NPDEEGLFTVAASVIIR<br>FPSTSESRNPDEEGLFTVAAS<br>VIIR<br>KATLHAVDVTLDPDTAHPH<br>LFLYEDSK	243	4	FPSTSESR<br>ATLVQDGIAK<br>EQEAEQMPEYR<br>NPDEEGLFTVAASVIIR			n.i.
Q14697	Neutral alpha-glucosidase AB	10687	5.74	363	3	EPWLLPSQHNDIIR<br>DVHNIYGLYVHMATADGL<br>R<br>FSFSGNTLVSSSADPEGHFET<br>PIWIER	66	1	VTEGGEPYR			n.i.
Q6WN34	Chordin-like protein 2 OS=Homo sapiens	49643	6.75	252	2	LPSPDPGAEGHGQSR<br>GPGTPAPTGLSAPLSFIPR	561	7	VTASPDKVTK<br>GIFHLTQIK<br>ADPGHSEISSTR<br>VLVHTSVSPSPDNLR<br>VLVHTSVSPSPDNLRR<br>FALEHEASDLVEIYLWK<br>LLAGPHEGHWNVFLAQTLEL<br>K	146	4	GAGSTTVKIVLK<br>LPSPDPGAEGHGQSR<br>SLERLPSPDPGAEGHGQSR<br>RGPGTPAPTGLSAPLSFIPR
Q96DA0	Zymogen granule protein 16 homolog B	22725	6.74	200	3	VSVGLLLVK<br>YFSTTEDYDHEITGLR<br>LGALGGNTQEVTLQPGEYIT<br>K	203	3	VSVGLLLVK<br>YFSTTEDYDHEITGLR<br>LGALGGNTQEVTLQPGEYITK			n.i.
Q96S86	Hyaluronan and proteoglycan link protein 3	40868	6.07	40	1	DLLNGVK	143	3	DLLNGVK<br>LTLTEAR<br>LSENGAPEKDVLVAIGLR			n.i.

Q99102	Isoform 10 of Mucin-4	225293	5.78	78	1	TVDFTSPLFKPATGFPLGSSLR	119	2	TVDFTSPLFKPATGFPLGSSLR<br>GTTFYQEYETFYGEHSLLVQQAESWIR	n.i.
Q9H173	Nucleotide exchange factor SIL1	52052	5.27	109	1	MFAEEEAELTQEMSPEK	73	2	VQVEAIEGGALQK<br>LLVILATEQPLTAK	n.i.

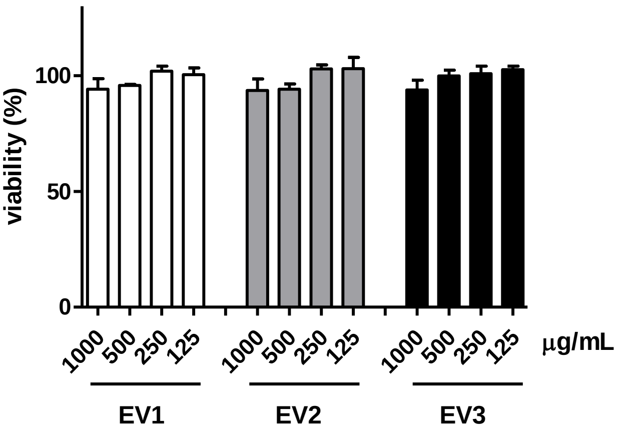

Figure S1. Cell viability assay. Evaluation of the effect of three EVs preparations (EV1, EV2 and EV3) on HFF-1 viability at 5 days. HFF-1 cells were treated under the same conditions as the antiviral assays and processed by means of an MTS assay. Data are reported as percentages of viable cells in comparison to the controls as determined by MTS assay for 1000, 500, 250, and 125 µg/mL concentrations. Three independent experiments were performed.
